# Stage- and Gender-Specific Proteomic Analysis of *Brugia malayi* Excretory-Secretory Products

**DOI:** 10.1371/journal.pntd.0000326

**Published:** 2008-10-29

**Authors:** Yovany Moreno, Timothy G. Geary

**Affiliations:** Institute of Parasitology, McGill University, Ste-Anne-de-Bellevue, Quebec, Canada; University of Pittsburgh, United States of America

## Abstract

**Introduction:**

While we lack a complete understanding of the molecular mechanisms by which parasites establish and achieve protection from host immune responses, it is accepted that many of these processes are mediated by products, primarily proteins, released from the parasite. Parasitic nematodes occur in different life stages and anatomical compartments within the host. Little is known about the composition and variability of products released at different developmental stages and their contribution to parasite survival and progression of the infection.

**Methodology/Principal Findings:**

To gain a deeper understanding on these aspects, we collected and analyzed through 1D-SDS PAGE and LC-MS/MS the Excretory-Secretory Products (ESP) of adult female, adult male and microfilariae of the filarial nematode *Brugia malayi*, one of the etiological agents of human lymphatic filariasis. This proteomic analysis led to the identification of 228 proteins. The list includes 76 proteins with unknown function as well as also proteins with potential immunoregulatory properties, such as protease inhibitors, cytokine homologues and carbohydrate-binding proteins. Larval and adult ESP differed in composition. Only 32 proteins were shared between all three stages/genders. Consistent with this observation, different gene ontology profiles were associated with the different ESP.

**Conclusions/Significance:**

A comparative analysis of the proteins released *in vitro* by different forms of a parasitic nematode dwelling in the same host is presented. The catalog of secreted proteins reflects different stage- and gender-specific related processes and different strategies of immune evasion, providing valuable insights on the contribution of each form of the parasite for establishing the host–parasite interaction.

## Author Summary

To succeed in infection, parasites must have ways to reach the host, penetrate its tissues and escape its defense systems. As they are not necessarily fatal, most helminth parasites remain viable within their host for many years, exerting a strong influence over the host immune function. Many of these functions are performed by products that are released from the parasite. We exploited the remarkable sensitivity of modern proteomics tools together with the availability of a sequenced genome to identify and compare the proteins released *in vitro* by adult males, adult females and the microfilariae of the filarial nematode *Brugia malayi*. This parasite is one of the etiological agents of lymphatic filariasis, a disease that poses continuing and significant threats to human health. The different forms of the parasite inhabit different compartments in the mammalian host. We found that the set of proteins released by each form is unique; they must reflect particular developmental processes and different strategies for evasion of host responses. The identification of these proteins will allow us to illuminate the biology of secretory processes in this organism and to establish a path for developing an understanding of how these parasite proteins function in immune evasion events.

## Introduction

Lymphatic filariasis (LF) is a disabling and disfiguring parasitic disease caused by the adult and developing forms of filarial nematode parasites residing in the lymphatic system of a mammalian host. The infection in humans is caused by *Wuchereria bancrofti*, *Brugia malayi* or *B. timori* [1] and puts at risk an estimated 1307 million people in 83 endemic countries in subtropical and tropical regions of the world [2].

Lymphatic filarial parasites have a two-host life cycle. Infection is initiated with the release by the mosquito of third stage larvae (L3) during feeding on the host. The L3 enter the host at the puncture site, penetrate the dermis and enter the lymphatic system. L3 parasites initiate a developmental program that culminates in a molt to fourth stage larvae (L4) 9–14 days post-infection. L4 undergo dramatic growth during the next 6 to 12 months as they develop into mature adults. Adult worms tend to localize in the varices of lymphatic vessels of the lower extremities. After insemination, zygotes develop *in utero* to microfilariae (Mf) over a three-week period. Adult female (F) parasites can remain reproductively active for >5 years. Females release hundreds to thousands of fully-formed, sheathed microfilariae per day into the lymphatic circulation of the host. From the lymph, they transit into the peripheral circulation.

Dramatic clinical manifestations, including hydrocoele, recurrent adenolymphangitis, lymphedema and elephantiasis are associated with chronic infection. Nevertheless, the majority of infected individuals have no clinically apparent sequelae, despite the presence of circulating Mf (and parasite antigens) in the peripheral blood [3]. Associated with the asymptomatic state is a suppression of both Th1 and Th2 responses, which may lead to high parasite loads and reduced immune-related damage to the host. This down-regulation of the host immune response is characterized by impaired proliferation of T cells, increased production of the regulatory cytokine IL-10, and higher levels of IgG4 [4].

The complexity of immune responses in LF is due, among other factors, to the presence of different life cycle stages of the parasite and the different levels of anatomical compartmentalization in which they reside [5],[6]. In addition, the presence in filarial nematodes of a *Wolbachia* endosymbiont, a matrilineally inherited obligate intracellular bacteria, contributes to this complexity, as *Wolbachia* antigens have been related to the development of inflammatory-mediated filarial disease [7]–[9].

While we lack a complete understanding of the molecular mechanisms by which pathogens achieve protection from host immune responses, it is generally accepted that parasitic nematodes release a variety of products, primarily proteins (many having posttranscriptional modifications), that enable infection by facilitating penetration of tissue barriers, migration through host tissues and evasion of immune responses. The characteristics and functions of these products are diverse and must reflect, among other factors, the lifestyle of each parasite. Even though their importance for establishing and maintaining the host-parasite interaction is accepted, relatively little is known about the mechanism(s) by which proteins secreted from nematodes regulate the immune system.

Proteins released from these parasites during culture *in vitro* are conventionally named excretory/secretory products (ESP). Several have been identified and characterized, particularly from *Brugia malayi*, an organism that can be maintained in the laboratory as a model of filarial nematodes and was chosen as a representative species to be analyzed for the Filarial Genome Project [10]–[12]. A draft of this genome was recently released [13], allowing the identification through proteomic analysis of the ESP from adult males (M) and females (F) of this parasite maintained together *in vitro* [14]. To gain a deeper understanding of how these parasites survive in their particular host milieu and the contribution of each form to the progression of the infection, we present a comparative analysis of the ESP independently released by Mf, F and M *B. malayi*.

## Materials and Methods

### Parasites

Mf and adult *B. malayi* were recovered >120 days post-infection from the peritoneal cavities of jirds (*Meriones unguiculatus*) infected subcutaneously with 200–300 L3. Infected jirds were obtained from the Filariasis Research Reagent Repository Center (Athens, Georgia USA). Adult worms were washed several times in RPMI 1640 medium [with L-glutamine, 20 mM HEPES, 100 µg/ml penicillin, 100 units/ml streptomycin (Gibco), pH 7.2] (henceforth, RPMI 1640) and separated by gender. Mf were obtained through several washes of the peritoneal cavity with 37°C RPMI 1640. The combined washes were centrifuged 5 min at 1000×g to pellet the Mf, which were subsequently purified from host cells by passage through PD-10 columns equilibrated with pre-warmed RPMI 1640 as described [15]. Animal procedures were reviewed and approved by the Facility Animal Care Committee of McGill University – Macdonald campus and were conducted in accordance with the guidelines of the Canadian Council on Animal Care.

### Parasite culture and preparation of ESP

Parasites were cultured in RPMI 1640 at 37°C for 4 days with changes of media each 24 hr [16]. F, M and Mf were maintained at densities of 6, 15 and 2.5×10^5^ parasites/ml, respectively. A cocktail of protease inhibitors [4-(2-aminoethyl)benzenesulfonyl fluoride hydrochloride; bestatin hydrochloride; N-(trans-epoxysuccinyl)-L-leucine 4-guanidinobutylamide; pepstatin A; phosphoramidon disodium salt] (Sigma No P8849, St. Louis, MO) was added to media containing ESP following sterilization by passage through a 0.22 µm filter. Media were stored at −30°C until analysis.

The combined volume (30–165 ml) of 3 different incubations was concentrated to 1–1.5 ml in an Amicon Ultra 3000 MWCO (Millipore). Proteins were then precipitated with Trichloroacetic acid (TCA, 20% final conc.). The pellet was washed 3 times with cold acetone (−30°C) and air-dried. Proteins were resuspended in Tris-HCl [20 mM, pH 8.0] and quantified (Quant-iT™ Protein Assay on a Qubit fluorimeter; Invitrogen).

### 1D electrophoresis and band excision

**Figure 1:**
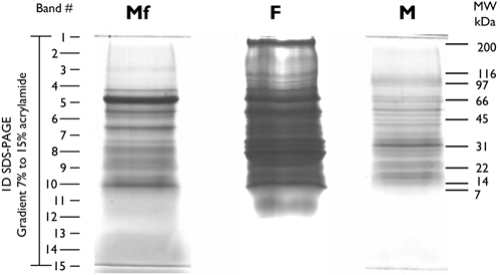
SDS-PAGE of ESP from microfilariae (Mf), females (F) and males (M) *B. malayi*. 37.3 µg, 65.3 µg and 15.4 µg of protein from Mf, F and M, respectively, were separated by electrophoresis through a 2.4 cm gradient SDS gel (7.5–14%). Protein loaded is the amount recovered from 3 pooled sets of independent incubations. Following staining with Coomassie Brilliant blue G, the entire lanes were subjected to automated band excision to obtain 15 pieces per lane. Proteins from gel bands were digested with trypsin and analyzed by LC-MS/MS. Intensity of lane F was adjusted in order to allow better visualization of the staining.

Concentrated ESP from Mf, F and M were centrifuged at 20000×g for 3 min and resuspended in loading buffer containing 2-mercaptoethanol. Final amounts of 37 µg, 65 µg and 15 µg of protein, respectively, were separated by SDS-PAGE on a 2.4 cm gradient gel (7–15% acrylamide). The gel was stained with Coomassie Brilliant blue G. The entire lanes were subjected to automated band excision, to generate 15 bands per lane (see Figure 1). The Protein Picking Workstation ProXCISION (Perkin Elmer) was set to excise 5 to 7 pieces per band, depending on the width of the lane.

### Tryptic digestion and Liquid Chromatography – Tandem Mass Spectrometry (LC-MS/MS) analysis

Proteins from gel bands (5 to 7 gel pieces per band/well) were subjected to reduction, cysteine-alkylation and in-gel tryptic digestion in a MassPrep Workstation (Micromass, Manchester, UK) as previously described [17]. Briefly, gel pieces were pre-washed twice in 100 µl HPLC grade water for 10 min. Gel pieces were destained in 2–10 min incubations in 50 µl 100 mM ammonium bicarbonate followed by 5 min in 50 µl 100% acetonitrile.

Destained and dehydrated gel pieces were reduced and alkylated by incubation in 50 µl 10 mM dithiothreitol for 30 min, followed by addition of 50 µl 55 mM iodoacetamide for 20 min and finally 100 µl 100% acetonitrile for 5 min. Gel pieces were washed by incubation for 10 min in 50 µl 100 mM ammonium bicarbonate, followed by a 5 min incubation in 50 µl 100% acetonitrile and were then dried for 30 min at 37°.

Proteins were digested in-gel by incubation in 25 µl trypsin solution (6 ng/µl in 50 mM ammonium bicarbonate, Promega) for 30 min at room temperature, followed by 4.5 hr at 37°C. Peptides were initially extracted with 30 µl of a mix containing 1% formic acid and 2% acetonitrile at room temperature and then by two successive extractions with 12 µl of a mix of 1% formic acid and 2% acetonitrile and 12 µl of 100% acetonitrile.

Using the Micro Well-plate sampler and the IsoPump modules of an an Agilent 1100 Series Nanoflow HPLC, 20 µl of the tryptic digest solution was injected on a Zorbax 300SB-C18 pre-column (5×0.3 mm, 5 µm) linked to an Agilent 1100 Series HPLC-system previously conditioned with water containing acetonitrile (3%) and formic acid (0.1%). The sample was washed for 5 min at 15 µl/min and subsequently the valve holding the pre-column was actuated to connect it between the NanoPump module and the flushed through a 75 µm ID PicoFrit column (New Objective, Woburn, MA) filled with 10 cm of BioBasic C18 (5 µm, 300 Å) in order to allow elution of the peptides towards the mass spectrometer at a flowrate of 200 nL/min. The acetonitrile concentration was first raised linearly from 10% to 40% in 40 min. It was increased linearly to 70% in 8 min, then to 95% in 5 min. The acetonitrile was held at 95% for 2 min then brought back to 10% in 2 min. The system was left at 10% acetronitrile for 3 min before starting the next injection. The total cycle time was 65 min. Eluted peptides were analyzed in a QTof micro (Waters Micromass, Manchester, UK)) equipped with a ZSpray Nanoflow stage modified with a ADPT-MZS nanospray adapter (New Objective, Woburn, MA). MS survey scan was set to 1 s (0.1 s interscan) and recorded from 350 to 1600 *m/z*. MS/MS scans were acquired from 50 to 1990 *m/z*, scan time was 1.35 s and the interscan interval was 0.15 s. The doubly and triply charged selected ions were selected for fragmentation with collision energies calculated using a linear curve from reference collision energies.

MS/MS raw data were transferred from the QTof micro computer to a 50 terabytes server and automatically manipulated for generation of peaklists by employing Distiller version 2.1.0 (http://www.matrixscience.com/distiller.htmls) software with peak picking parameters set at 30 as for Signal Noise Ratio (SNR) and at 0.6 for Correlation Threshold (CT). The peaklisted data were searched against a copy of the Universal Protein Resource (UniProt) data base (March 03, 2008) by employing Mascot (http://www.matrixscience.com) version 2.1.04, and restricting the search to up to 1 missed (trypsin) cleavage, fixed carbamidomethyl alkylation of cysteines, variable oxidation of methionine, 0.5 mass unit tolerance on parent and fragment ions, and monoisotopic. The search was limited to the *Brugia* and *Wolbachia* taxonomies (Taxonomy ID: 6278 and 953, respectively) (17537 sequences; 5684760 residues).

Mascot results from bands 1 to 15 (based on spectra assigned to tryptic peptide sequences at the 95% confidence level) generated peptide identifications that were then linked to the proteins and sorted by protein to produce an initial list of protein identifications. The list was quite redundant since about 5% of the spectra matched more than one peptide and 40% of the peptides identified occur in more than one protein. Consequently, the sequences were processed by a grouping algorithm [18] to generate a list of proteins defined by distinct sets of proteins. That is, the minimum number of protein sequences needed to explain the peptides observed. This minimal list of proteins was summarized on a SubGroup Count Report.

### Bioinformatics

Sequences from the minimal list of proteins were retrieved from the UniProt database and scanned for prediction of signal peptides and subcellular localization with SignalP 3.0 [19], TargetP 1.1 [20] and SecretomeP 2.0 [21].

### Gene Ontology (GO)

GO annotations were performed using Blast2GO [22]. The initial Blastp search was performed against the NCBI nonredundant database with a minimum expectation value of 1×10^−3^ and a high scoring segment pair cut-off of 33. Annotations were made with default parameters; the pre-eValue-Hit-Filter was 1×10^−6^, Annotation cut-off was 55, and GO Weight was 5. Annotation was augmented by using the Annotation Expander (ANNEX) [23] and by addition of the GO terms associated with functional domains derived from scanning the InterPro database [24],[25].

The statistical framework GOSSIP [26] was used to identify statistically enriched GO terms associated with gender- or stage-specific secreted proteins compared to the GO terms associated with the complete set of identified proteins. Contingency tables for each GO term in the test group were generated and P values calculated in a Fisher's exact test. The P values were adjusted for multiple testing by calculation of the false discovery rate and the family wise error rate.

## Results

### Protein identification

ESP were obtained from F, M and Mf under previously described conditions [16],[27],[28]. Average protein recoveries from several incubations were 71 and 41 ng protein per worm per day for F and M, respectively, and 165 ng protein per 1×10^6^ Mf per day, values that are in the same order of magnitude as reported by others [16],[27]. No remarkable changes in motility or physical integrity of the worms were observed during the 4 day incubation, suggesting that the collected media contained the products of normal physiological activity of the parasites and not the products of death or leaking caused by the procedures.

The analysis of ESP from parasitic nematodes is limited by the very low amount of protein usually recovered from *in vitro* incubations. We addressed this issue by comparing several techniques for concentration and desalting at small scale. Although trichloroacetic acid (TCA) precipitation gave higher yields of protein than Amicon (MWCO 3000) ultrafiltration, scaling the TCA precipitation up to 20 ml caused the retention of a significant amount of salt in the preparation. This generated wide lane profiles on SDS-PAGE, resulting in dilution of the proteins in the gel and diminishing the chances for identification. A preliminary analysis under these conditions led to the identification of 15 proteins in F, M, and Mf preparations, which ranged in amount from 6.5 to 13 µg protein (not shown).

As an alternative, we concentrated and desalted ESP by using Amicon devices to attain a final volume of 1–1.5 ml, and then precipitated the proteins with TCA. Preparations obtained in this way provided narrower running profiles with no apparent signs of proteolytic degradation (Figure 1). For this analysis, we pooled and concentrated the total media recovered from 3 sets of independent incubations. One set of incubations corresponds to the total number of worms and larvae recovered from one infected animal. This procedure led to the recovery of 37.3 µg, 65.3 µg and 15.4 µg of protein for Mf, F and M, respectively, subsequently subjected to SDS-PAGE (Figure 1).

For protein identification, each lane of a gradient 2.4 cm SDS-PAGE gel was excised, digested with trypsin and analyzed by Liquid Chromatography – Tandem Mass Spectrometry (LC-MS/MS). Initial peptide matches led to the preliminary identification of 286 proteins in the total ESP set. Refinement of the assignment of peptides to authentic proteins as described in the Methods section. This approach allowed us to identify 228 distinct proteins as part of the ESP of Mf, F and M (Table 1 and S1). All except one, annotated as Mmc-1(UniProt ID: Q9NDV4), were matched to proteins annotated in the *B. malayi* genome database. Peptides identified in the MS/MS analysis were assigned with high confidence to *B. pahangi* Mmc-1, which was also previously identified in Mf-ESP from this parasite [29]. A homolog nucleic acid sequence for the *B. pahangi* Mmc-1gene is also present in the *B. malayi* EST data set (Gene Index: TC11321). Its absence at the moment from the *B. malayi* protein models likely reflects the incomplete assembly available in the current version of the database and not a false-positive assignment.

**Table 1:** Most abundant proteins identified in the ESP of *B. malayi*.

		Uniprot ID	Pub_locus	TIGR locus	DESCRIPTION	SS	SecP	NQPCT		
								Mf	Fw	Mw
**Microfilariae**	1	A8NW22	Bm1_11105	13673.m00035	Recombinant antigen R1, identical	Y	0.70	14.35	0.27	0.15
	2	P29030	Bm1_17035	14274.m00229	Endochitinase precursor	Y	0.53	13.04	0.00	0.00
	3	A8PJW0	Bm1_28525	14931.m00318	Serpin	Y	0.49	9.42	0.00	0.00
	4	A8Q2C4	Bm1_41005	14973.m02599	OV-16 antigen, putative	N	0.60	4.64	0.75	1.83
	5	A8QEB1	Bm1_51000	14992.m10974	Putative uncharacterized protein	N	0.51	4.20	5.19	5.55
	6	A8NQM6	Bm1_07780	13333.m00082	Immunogenic protein 3, putative	Y	0.88	3.91	0.98	1.32
	7	A8PKM4	Bm1_29130	14940.m00172	Triosephosphate isomerase, putative	N	0.36	3.48	16.87	3.73
	8	O97392	Bm1_09950	13531.m00015	Gamma-glutamyl transpeptidase precursor	Y	0.69	2.75	3.35	0.81
	9	A8P664	Bm1_17400	14293.m00075	Trypsin inhibitor, putative	N	0.69	2.61	0.00	0.00
	10	A8PGM6	Bm1_24940	14731.m01012	Galectin, putative	N	0.35	2.61	5.22	0.56
	11	A8PVV9	Bm1_35870	14971.m02814	Copper type II ascorbate-dependent monooxygenase, C-terminal domain containing protein	N	0.65	2.03	0.00	0.00
	12	Q9BJC9	-	-	Major allergen	Y	0.76	2.03	1.69	1.02
	13	A8QFZ3	Bm1_54345	15148.m00017	Zinc finger, C2H2 type family protein	N	0.55	1.80	0.00	0.00
	14	O16159	Bm1_56600	15418.m00009	Cystatin-type cysteine proteinase inhibitor	Y	0.96	1.74	1.83	1.39
	15	Q6T8C4	-	-	Superoxide dismutase [Cu-Zn]	N	0.39	1.74	0.58	1.17
**Female worm**	1	A8PKM4	Bm1_29130	14940.m00172	Triosephosphate isomerase, putative	N	0.36	3.48	16.87	3.73
	2	A8PJU3	Bm1_28435	14930.m00337	Bm-MIF-1, identical	N	0.51	0.72	8.91	0.15
	3	A8QH34	Bm1_56305	15373.m00009	Leucyl aminopeptidase, putative	Y	0.86	0.58	7.72	1.83
	4	A8QFI4	Bm1_53510	15059.m00091	Myotactin form B, putative	N	0.35	0.00	5.24	7.17
	5	A8PGM6	Bm1_24940	14731.m01012	Galectin, putative	N	0.35	2.61	5.22	0.56
	6	A8QEB1	Bm1_51000	14992.m10974	Putative uncharacterized protein	N	0.51	4.20	5.19	5.55
	7	O97392	Bm1_09950	13531.m00015	Gamma-glutamyl transpeptidase precursor	Y	0.69	2.75	3.35	0.81
	8	P67877	Bm1_40465	14972.m07803	Cuticular glutathione peroxidase precursor	Y	0.59	0.29	2.74	7.69
	9	A8PFE3	Bm1_24115	14703.m00079	Enolase, putative	N	0.53	1.16	2.54	4.69
	10	O16159	Bm1_56600	15418.m00009	Cystatin-type cysteine proteinase inhibitor	Y	0.96	1.74	1.83	1.39
	11	Q9BJC9	-	-	Major allergen	Y	0.76	2.03	1.69	1.02
	12	A8Q119	Bm1_40580	14972.m07829	Glycosyl hydrolases family 31 protein	N	0.59	0.00	1.49	1.39
	13	A8Q0F4	Bm1_40185	14972.m07743	Calsequestrin family protein	Y	0.76	0.00	1.32	0.22
	14	A8P3E5	Bm1_15350	14176.m00093	Fructose-bisphosphate aldolase 1, putative	N	0.40	0.00	1.29	6.00
	15	A8QEM1	Bm1_51495	14992.m11078	Heat shock protein 90, putative	N	0.18	0.00	1.08	0.44
**Male worm**	1	P67877	Bm1_40465	14972.m07803	Cuticular glutathione peroxidase precursor	Y	0.59	0.29	2.74	7.69
	2	A8QFI4	Bm1_53510	15059.m00091	Myotactin form B, putative	N	0.35	0.00	5.24	7.17
	3	A8P3E5	Bm1_15350	14176.m00093	Fructose-bisphosphate aldolase 1, putative	N	0.40	0.00	1.29	6.00
	4	A8NPW6	Bm1_07275	13311.m00333	Core-2/I-Branching enzyme family protein	N	0.67	0.00	0.10	5.68
	5	A8QEB1	Bm1_51000	14992.m10974	Putative uncharacterized protein	N	0.51	4.20	5.19	5.55
	6	A8PFE3	Bm1_24115	14703.m00079	Enolase, putative	N	0.53	1.16	2.54	4.69
	7	A8P0Q6	Bm1_13605	14015.m00091	Major Sperm Protein	N	0.62	0.00	0.38	4.63
	8	A8PKM4	Bm1_29130	14940.m00172	Triosephosphate isomerase, putative	N	0.36	3.48	16.87	3.73
	9	A8NLB0	Bm1_04870	13066.m00251	Putative uncharacterized protein	N	0.78	0.00	0.10	3.18
	10	A8NZQ9	Bm1_12945	13929.m00009	Lethal protein 805, isoform d, putative	Y	0.60	0.00	0.37	2.53
	11	Q4VWF8	Bm1_04665	13047.m00009	Independent phosphoglycerate mutase isoform 1	N	0.49	0.29	0.58	2.42
	12	A9XG48	Bm1_50995	14992.m10973	L3R15 repetitive antigen	N	0.63	0.00	1.04	1.85
	13	A8Q2C4	Bm1_41005	14973.m02599	OV-16 antigen, putative	N	0.60	4.64	0.75	1.83
	14	A8QH34	Bm1_56305	15373.m00009	Leucyl aminopeptidase, putative	Y	0.86	0.58	7.72	1.83
	15	A8P0Q4	Bm1_13600	14015.m00090	Major sperm protein 2 , putative cytoskeletal MSP	N	0.75	0.00	0.13	1.74

**SS:** Signal Peptide Prediction from SignalP. **SecP:** SecretomeP score; values >0.5 not having predicted Signal Sequence predict the possibility of non-classical secretion in mammalian cells. **NQPCT:** Prorated Query Count Percentage values.

### Comparison with previous ESP analyses

Many proteins have been identified as being secreted by *B. malayi* and related filarial nematodes by more traditional biochemical techniques (Table S2). All but two of the proteins reported to be secreted by *B. malayi* were found in the current survey. Neither of these two (acetylcholinesterase and Transforming growth factor - 2) were reported in the initial proteomic analysis [14]. Proteins reported to be secreted by other filariae all had homologues in the *B. malayi* ESP set.

This set included 59 of the 80 proteins (73.8%) reported in [14] from incubations of M and F worms in joint culture. Proteins not identified in the current study included 8 assigned in [14] through screening of the *B. malayi* EST database (Gene Index: TC7940, AI783143, TC9625, TC7985, TC8258, TC8116, TC7986 and AA592049). None of these is found in the current assembly of the *B. malayi* genome database, suggesting that further analysis may alter the assignment of the peptides to a genomic locus. In addition, nuclear function associated proteins (Pub locus: Bm1_03115, Bm1_46120, Bm1_25620), several with undetermined function (Bm1_19065, Bm1_57465, Bm1_46475, Bm1_11505, Bm1_01245, Bm1_09845 and Bm1_33310), 6-phosphofructokinase (Bm1_01930), a tropomyosin family protein (Bm1_02060) and phosphatidylethanolamine-binding protein 2 (Bm1_31500) were identified as relatively low abundance ESP proteins in [14] but were not detected in the current study. As reported in [14], none of the proteins detected in *B. malayi* ESP in the current study could be assigned to *Wolbachia* proteins.

According to the Prorated Query Count percentage values (NQPCT), which provide a measure of relative abundance of a protein in a sample [18], we found triosephosphate isomerase (TPI) to be one of the most abundant proteins in the ESP of all gender/stages, indeed the most abundant in F-ESP, consistent with other observations [14]. Although results from our separate incubations of M ad F worms generally agreed with those from the combined culture [14], we found some differences. A fasciclin domain containing protein (UniProt ID: A8P605) and an endochitinase (P29030) were exclusively associated with Mf-ESP and were not present in ESP from adults. These two proteins have relatively high NQPCT values in the Mf-ESP sample and, therefore, their identification as low abundance ESP proteins in [14] may be related to release of Mf in adult worm cultures.

62 (27.2%) of the identified proteins were predicted by SignalP [19] to have an amino-terminal secretion signal peptide and therefore may be secreted through the classical pathway. This value represents an enrichment of 11.8% in comparison to the proportion of total gene models in the *B. malayi* database having a predicted signal peptide. In addition, 81 (35.5%) of these proteins may be secreted through non-classical secretory pathways, as they were identified by SecretomeP [21] to share features with mammalian proteins secreted in this manner. The proportion of proteins bearing a secretion motif is similar in the current study compared to the proteins identified in [14], suggesting that the presence in ESP of proteins lacking known secretion motifs is not an artifact.

### Mf, M and F worms secrete different sets of proteins

**Figure 2:**
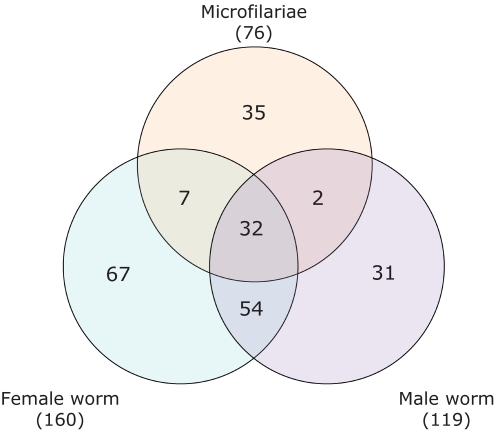
Venn diagram showing the distribution of proteins identified in ESP from microfilariae, female and male *B. malayi*.

76 proteins were identified in Mf-ESP. 160 and 119 were identified in F and M-ESP sets, respectively. Only 32 proteins (14.0%) were shared by all three stages/genders (Figure 2). Approximately half of these proteins had no annotated function or were poorly annotated (assigned a named match but with no associated functional domain or Gene Ontology term). F and M shared 54 proteins (23.7%), whereas Mf showed a much lower degree of similarity with the adult stages, with only 7 (3.1%) and 2 (0.9%) proteins shared with F and M-ESP, respectively.

Differences expressed as presence/absence of a protein can be extended to protein abundance. Table 1 presents a list of the 15 most abundant proteins as determined by NQPCT in each ESP. Most of the Mf-ESP proteins presented in this table were only identified at this stage or were not found as highly abundant proteins in ESP from adults. Differences in ESP composition between M and F were also observed; notable among these are the presence of Major Sperm Protein (MSP) family proteins as highly abundant in M-ESP, the appearance of a homolog of the human macrophage migration inhibitory factor (MIF-1, A8PJU3) as an abundant protein in F-ESP but not in M-ESP and numerous differences in the relative abundances of other proteins between the sexes.

### GO Analysis

We used the Blast2Go analysis tool to mine the GO based data to illuminate the different functions and processes in which the proteins identified in the ESP are putatively involved, The initial annotation was augmented by using the Annotation Expander (ANNEX) [23] and by addition of the GO terms associated with functional domains resulting from scanning the InterPro database [24]. This analysis provided ≥1 GO terms for 171 sequences (75%) from the total set. Of these, 157 (68.8% of the total) sequences could be assigned to terms associated with molecular functions and 136 (59.6% of the total) with biological functions.

**Figure 3:**
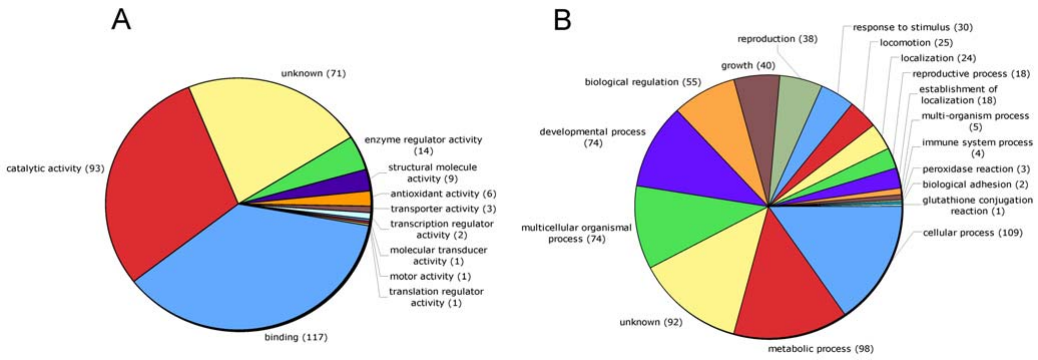
Distribution of Gene Ontology terms (level 2) for proteins identified in ESP from microfilariae, female and male *B. malayi*. A. Molecular Function. B. Biological Process.

**Figure 4:**
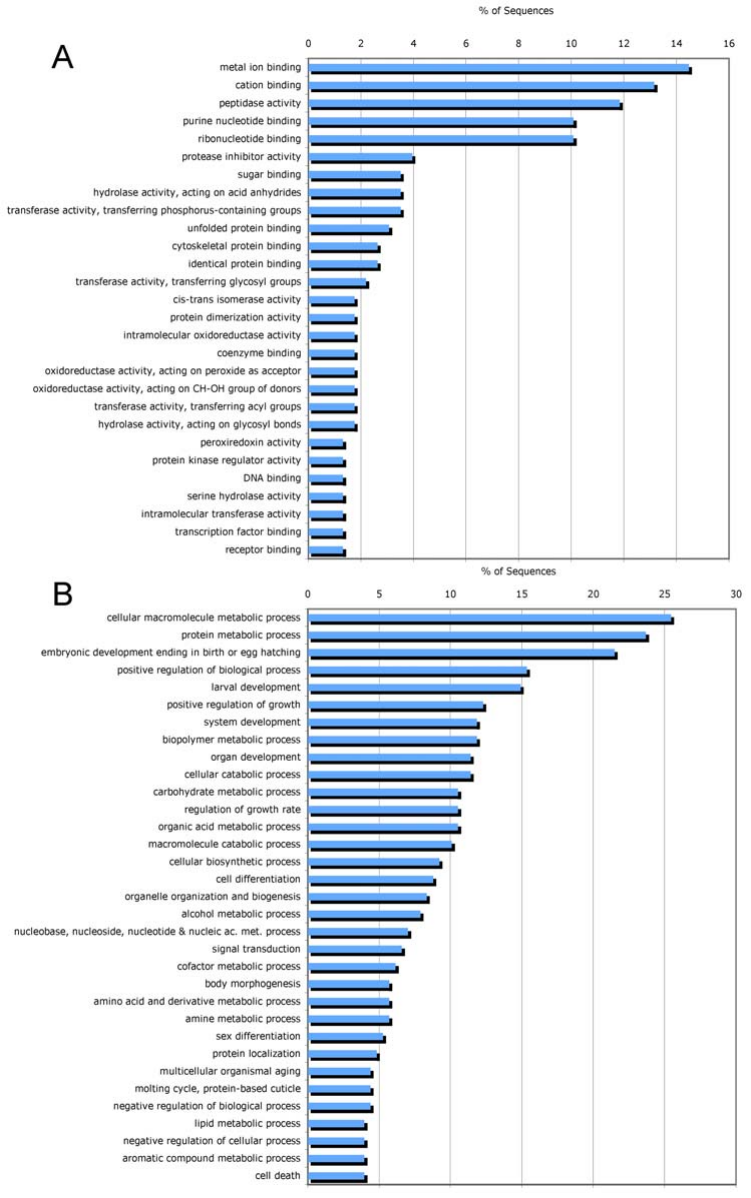
Distribution of the most abundant Gene Ontology terms (level 4) assigned for proteins identified in ESP from microfilariae, female and male worms of *B. malayi*. A. Molecular Function. B. Biological Process.

In the total set of ESP and the individual subsets (Mf, F and M), catalytic activity (GO:0003824) and binding (GO:0005488) were the two major molecular function categories (Figure 3A). Other molecular function categories include enzyme regulator activity (GO:0030234) and antioxidant activity (GO:0016209). Structural molecule activity (GO:0005198) was not found in Mf-ESP but was common in M-ESP (Figure S1). At a higher level of ontology (Figure 4A), most of the assigned binding activity could be assigned to metal ion binding and cation binding (GO:0046872 and GO:0043169), purine nucleotide binding (GO:0017076), ribonucleotide binding (GO:0032553) and, to a lesser extent, sugar binding (GO:0005529) and several terms related to protein binding (GO:0051082, GO:0008092 and GO:0042802). The catalytic activity function is populated by diverse types of reactions, with a major contribution from peptidase activity (GO:0008233). Interestingly, the protease inhibitor activity term (GO:0030414) was the most common in the enzyme regulator activity category.

The most common biological function categories (Figure 3B) were cellular process (GO: 0009987), metabolic process, multicellular organism process (GO:0032501), developmental process (GO:0065007) and, less commonly, biological regulation (GO:0065007), growth (GO:0040007) and reproduction (GO:0000003). As with the molecular function classes, proteins in these categories were found in the total set and individual subsets of ESP (Figure S2). In addition and as expected, proteins associated with reproduction (GO:0000003) were predominantly found in adult ESP.

A higher level of ontology (Figure 4B) shows that most of the cellular and metabolic processes are related to synthesis and degradation of macromolecules, particularly proteins and carbohydrates (GO:0044260, GO:0019538, GO:0043283, GO:0005975, GO:0009057), whereas the largest contribution to the multicellular organism and developmental processes came from terms such as embryonic development ending in birth or egg hatching (GO:0009792) and larval development (GO:0002164) that can be associated with the release and development of Mf.

**Figure 5:**
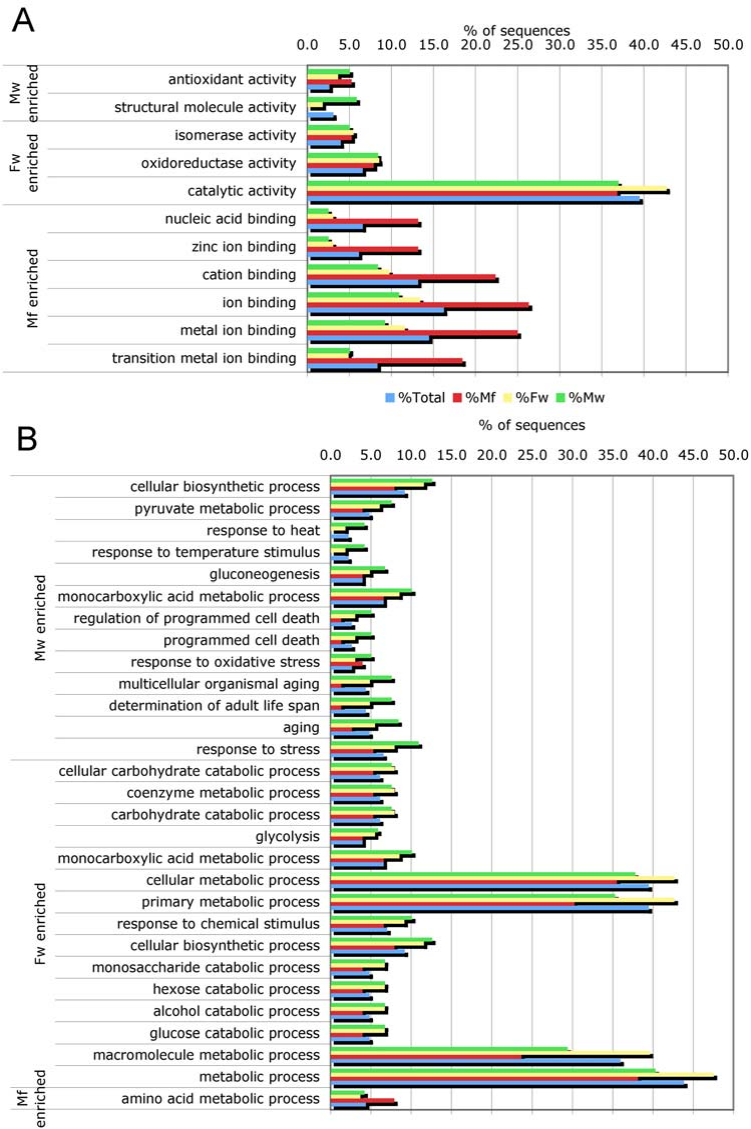
Gene Ontology terms concentrated on individual ESP from microfilariae, female and male *B. malayi* compared to the total protein set. A. Enriched molecular function terms. B. Enriched biological process terms.

The GOSSIP statistical framework [26] was used to determine the enrichment of particular functions or processes in the ESP from Mf, M and F. We compared the terms associated with the proteins identified in each of the 3 ESP sets against those from the total GO term annotated proteins. Several processes and functions had significant *P* values (*P*<0.05) in the single test (Figure 5). Nevertheless, to correct for multiple testing, a more stringent comparison using both a false discovery rate and a family-wise error rate was performed, and only the terms in the Mf-ESP were found to be enriched. These terms are all children of the parent ion binding term (GO:0043167), with zinc ion binding (GO:0008270) the term with the highest level of ontology.

## Discussion

Filarial infections pose continuing and significant threats to human and animal health. Although drugs such as ivermectin, diethylcarbamazine (DEC) and albendazole are currently used to interrupt disease transmission and reduce morbidity [30], there are concerns about the emergence of resistance for these drugs [31],[32]. Secreted products are thought to be essential for the establishment of the parasitic lifestyle and therefore their identification in filarial nematodes may lead to the discovery of novel drug and vaccine targets [33]–[36]. Moreover, their recognition will help to illuminate the biology of secretory processes in these organisms and to establish a path for developing a deeper understanding of how parasite proteins function in immune evasion.

We exploited the remarkable sensitivity of mass spectrometry and the availability of a genome with >11,500 predicted gene models [13] to identify proteins secreted *in vitro* by *B. malayi*. It is reasonable to assume that the profile and function of secreted proteins will vary with developmental stage and sex, and may also vary in response to signals from the host.

To begin an investigation into this area, we analyzed ESP from independently incubated Mf, M and F worms; this analysis thus extends in a new way the previous work done on ESP obtained from adult male and female *B. malayi* in co-culture [14]. In addition to the identification of 148 novel secreted proteins from filarial nematodes, the approach employed here allowed us to compare the ESP composition for each group. As we recognize in global terms these differences in the ESP from the different forms of a parasitic nematode, it should enable us to start to infer the different roles and processes in which these proteins are involved, in particular to gain a better understanding of how filarial parasites orchestrate immune evasion. This will open a new dimension in the understanding of the biological significance of protein secretion by parasitic nematodes.

Until recently, all previous work on the identification of ESP from filarial nematodes was performed by the study of a few, primarily immunoreactive, proteins. Comprehensive analyses of ESP are still limited by the low amounts of proteins that can be recovered from *in vitro* incubations of living worms. In this context, the use of 1D-SDS PAGE and LC-MS/MS may be a more efficient way to identify protein sequences in filarial ESP compared to the shotgun LC–MS/MS approach or the excision of particular spots from 2D-gels followed by MALDI-ToF/ToF [14]. This technical approach, in conjunction with homology-based analyses for mining of functional information on each of the hits, allows for a more complete understanding of the probable events and functions in which these components may be involved.

Effective prediction of ESP through the identification of signal sequences or other domain features is not feasible because, as we show here, many of the proteins released in culture would have been missed by secretome prediction tools, indicating that the processes involved in the release of these components are not fully understood. This issue, along with the need to identify non-protein components and post-transcriptional modifications of proteins in ESP that may have functional and essential roles, remain goals for the development of new tools to understand the basic biology of filarial and other parasitic infections.

### Proteins common to all stages/genders

About 14% of secreted proteins were found in all stages/genders. One can speculate that this group is likely to include proteins that are essential for survival in the host, for instance, by deflecting the immune response. However, a significant portion of the common ESP proteins have no annotated function or GO term that can help in inferring their roles; therefore, the challenge is to elucidate their possible functions as determinants of parasitism. This includes proteins putatively assigned to the transthyrethin-like family, recently reported in [14], a set of hypothetical proteins and previously identified but functionally uncharacterized antigens.

An intriguing aspect of the common ESP set is the prominent presence of the glycolytic enzymes enolase (A8PFE3) and TPI (A8PKM4). Although this finding could be due to death or compromised integrity of the parasites in culture, we believe this is not the case. First, other glycolytic enzymes, at least as abundant in cytoplasm as these two, do not appear in ESP. Secondly, these proteins have been previously identified in ESP from *B. malayi* and other parasitic nematodes [14],[35],[37],[38]. Finally, evidence has appeared about their multiple roles and interaction with surface components in eukaryotic cells; an extracellular role thus cannot be excluded [39]–[41].

Other proteins identified in all 3 stages/genders include potentially immunomodulatory proteins, including a homolog of MIF-1 (A8PJU3) [42], a galectin (GAL-1, A8PGM6), a cystatin-type cysteine proteinase inhibitor (CPI-2, O16159) [43] and leucyl aminopeptidase (LAP, A8QH34), a homolog of an ESP product that in *Acanthocheilonema viteae* (ES-62) is modified with N-Type glycans containing phosphorylcholine (PC) [44]. In contrast, the protein identified in [14] as harboring the PC moiety in *B. malayi*, a core-2/I-branching enzyme family protein (A8NPW6), was only found in adult stages together with another isoform of human MIF (MIF-2) (Q9NAS2) [45] and GAL-2 [14].

### ESP from Mf

Taking into account the number of proteins identified, their abundance and the possible functions and processes in which they can be predicted to be involved, it is clear that the composition of ESP from Mf is quite distinct from adult ESP. This result is perhaps unsurprising, since adults and larvae may exhibit different physiological repertoires reflecting their different developmental stage and anatomical location (blood vs. lymph). Differences in ESP composition also suggest that there may be differences in the mechanisms of immune evasion between the stages.

Several proteins with no assigned function were identified in Mf-ESP, including the protein identified as antigen R1 (A8NW22), which was the most abundant. This protein seems to be preferentially expressed in Mf, although it was also identified in ESP from M and F. Although no function is annotated for this protein, recombinant R1 has been used in diagnostic IgG4 ELISAs with excellent success for the detection of *B. malayi* infection using serum from Mf(+) patients, with however significant positivity in Mf(−) patients [46].

Many Mf-ESP proteins with predicted function are associated with developmental processes and regulation of enzyme activity. Some may play a role in the immunology of the host-parasite relationship, including an endochitinase (P29030) and a serpin (A8PJW0), which were the next most abundant proteins in Mf-ESP; both were only found at this developmental stage. Chitinases are essential for chitin degradation during molting of larval filariae [35]. P29030 was originally reported as an antigen recognized by MF1, a monoclonal antibody that mediates the transient clearance of Mf in jirds infected with *B. malayi* [47]. Immunization of jirds with recombinant endochitinase induced partial protection against Mf, but did not reduce adult worm burdens, suggesting that this protein is crucial for Mf development but not for adult viability [48].

GO analysis revealed that protease inhibition was the most common functional class in the ‘regulation of enzyme activity’ category. Mf secreted a completely different set of protease inhibitors than adults. In particular, homologues of serine protease inhibitors (SPI) were abundant in Mf-ESP. This set included serpins, a class of proteins having a wide spectrum of functions at extracellular and intracellular levels in eukaryotic cells [49]. In mammals, serpins are involved in the regulation of fundamental processes, including coagulation, complement activation and inflammation [50],[51], but their potential roles as modulators of host responses in lymphatic filariasis are uncertain [52]. The serpin identified as A8PJW0 is the predicted gene model for *B. malayi* serine protease inhibitor 2 (Bm-SPN-2, UniProt Accession No. O18656). Bm-SPN-2 induces a Th1 response as characterized by the *in vitro* production of IFN-γ but not IL-4 or IL-5 in murine T cells [53]. At least 14 serpins are predicted in the *B. malayi* genome, two of which (A8PHV4 and A8PHV1) were present in lower abundance compared to Bm-SPN2, and both of them only in Mf-ESP.

In addition to serpins, another abundant serine protease inhibitor in Mf-ESP is a putative trypsin inhibitor (A8P664). A8P664 contains a trypsin inhibitor-like cysteine rich domain (TIL) that can potentially inhibit peptidases belonging to families S1, S8, and M4. A8P664 shares 50% identity with a trypsin inhibitor from the gastrointestinal nematode *Ascaris suum*, but is less related to serine protease inhibitors from the filarial nematodes *Dirofilaria immitis* (Di-SPI-1) and *Onchocerca volvulus* (Ov-SPI-1, Ov-SPI-2) [54]. Bm-SPI-1, another inhibitor from *B. malayi* [54], was not found in any of the ESP and has no significant homology with A8P664. In *A. suum* and other gastrointestinal nematodes, secretion of trypsin inhibitors has been proposed to interfere with the action of host digestive enzymes and with immunological effector mechanisms [55]–[58]. The Ov-SPI proteins seem to play a crucial role in nematode molting and in processes such as embryogenesis and spermatogenesis [54]. The roles of A8P664 may be different, as it is only found in Mf-ESP.

Another potential serine protease inhibitor present in relatively high abundance in adult and Mf-ESP is a homolog of the *O. volvulus* antigen Ov16, identified as A8Q2C4 [59]. A8Q2C4 was also identified in [14] in adult ESP as a homolog of proteins in the phosphatidylethanolamine-binding protein (PEBP) family, including a secreted 26-kDa antigen from the ascarid *Toxocara canis* [60] and a mouse PEBP with inhibitory activity against several serine proteases [61].

An intriguing finding in Mf-ESP is the presence of several zinc finger (ZnF) C2H2- type family proteins (A8QFZ3, A8PEN7, A8PLP4, A8QHJ5, A8QHP5, A8ND91). These proteins were classified in the GO analysis with the Zinc Ion binding term, a molecular function that was enriched in Mf-ESP compared to the total protein set of ESP. The most common role assigned to ZnF proteins is the control of gene transcription through binding to specific DNA segments [62]. In addition, ZnF motifs mediate RNA, protein and lipid binding [63],[64]. Although no apparent function has been assigned to these nematode proteins as mediators of extracellular processes, the fact that all of them were found in a particular stage with relatively high levels of secretion in comparison to other known secreted proteins deserves further consideration.

### Possible differences between Mf and adults in oxidative stress and nitric oxide evasion

Filarial nematodes deploy several mechanisms to detoxify reactive oxygen and nitric oxide derivatives produced by the host [65]. Based on our results, the different stages may use different mechanisms to overcome this type of stress. For example, gp29 (P67877), a glutathione peroxidase believed to act as a lipid hydroperoxidase, protecting parasite membranes from peroxidation caused by oxygen free radicals [66]–[68], was identified in all 3 ESP sets. However, the markedly higher abundance of gp29 in adult compared to Mf ESP suggests that this enzyme may play a more important role in adult survival compared to Mf. In contrast, γ-glutamyl transpeptidase (γ-GT, O97392) [69], glutathione S-transferase (GST, A8Q729) [70] and Zn-Cu superoxide dismutase (SOD, Q6T8C4) [71]–[73] appeared with similar abundance in ESP from all three gender/stages of the parasite. Thus, mammalian-stage parasites may use common strategies to defuse free radicals in the host microenvironment by modulating the levels of glutathione, or by using the thioredoxin (TRX-1, A8Q921) system as a source of reducing equivalents [74],[75]. In addition to its role in transferring the γ-glutamyl moiety of glutathione to an acceptor [76], *B. malayi* γ-GT can trigger autoimmunity against human γ-GT in lung epithelial cells and may play a role in the development of the local pulmonary pathology syndrome known as Tropical Pulmonary Eosinophilia [77].

### Sex-specific secreted proteins

Several sex-specific secreted proteins were identified in this study, characterized by relatively low abundance. This list may be underpopulated due to the fact that some of the proteins identified in both stages could be misclassified as the result of transferring of proteins from male to females worms during insemination previous to the recovery of worms from the jird host, as is likely the case for major sperm protein 2 (MSP-2), expressed only in M [78] but found in both M and F-ESP. Other members of the MSP family were found only in M-ESP. In male *B. malayi* and other nematodes, MSP are expressed in the developing sperm and reproductive system and are essential for nematode sperm motility [79],[80]. It is possible that proteins exclusively identified in F-ESP are released in uterine fluid, as some of them including the embryonic fatty acid binding protein (Bm-FAB-1, Q9GU91) and the papain family cystein protease containing protein (A8NND7) have been reported to be expressed in the uterus as well as in developing gametes and embryos [79].

### Conclusions

This proteomic analysis led to the identification of multiple components of the ESP of Mf, F and M *B. malayi*. In addition to the report of new identified ESP, the opportunity to compare the composition of ESP in all three stages/genders allowed us to propose different stage and gender specific related processes and to identify candidate proteins that may underlie stage specific strategies of immune evasion.

## Supporting Information

Figure S1 Distribution of Molecular Function Gene Ontology terms (level 2) compared between stages/genders of *B. malayi*. A. total set of ESP identified, B. ESP identified in microfilariae, C. ESP identified in females, D. ESP identified in males.
(1.86 MB TIF)

Figure S2 Distribution of Biological Process Gene Ontology terms (level 2) compared between stages/genders of *B. malayi*. A. total set of ESP identified, B. ESP identified in microfilariae, C. ESP identified in females, D. ESP identified in males.
(2.39 MB TIF)

Table S1 Proteins identified in ESP from microfilaria, female and male *B. malayi*. TP: Prediction of subcellular localization by TargetP, (M): Mitochondrion, (S): Secretory pathway (−): Any other location. RC: Reliability class, from 1 to 5, where 1 indicates the strongest prediction. GO: Gene Ontology. EC: Enzyme Commission number. NQPCT: Prorated Query Count Percentage values.
(0.78 MB DOC)

Table S2 List of previously secreted proteins identified by other methods in *B. malayi* and other parasitic nematode species.
(0.11 MB DOC)

Alternative Language Abstract S1 Translation of the Abstract into Spanish by Yovany Moreno
(0.01 MB PDF)

Alternative Language Abstract S2 Translation of the Abstract into French by Yovany Moreno and Timothy G. Geary
(0.01 MB PDF)
