# Supplementary figures and images for: Stage- and Gender-Specific Proteomic Analysis of Brugia malayi Excretory-Secretory Products
Source: PLoS Negl Trop Dis. 2008 Oct 29;2(10):e326. doi: 10.1371/journal.pntd.0000326 (PMC2569413; doi:10.1371/journal.pntd.0000326)

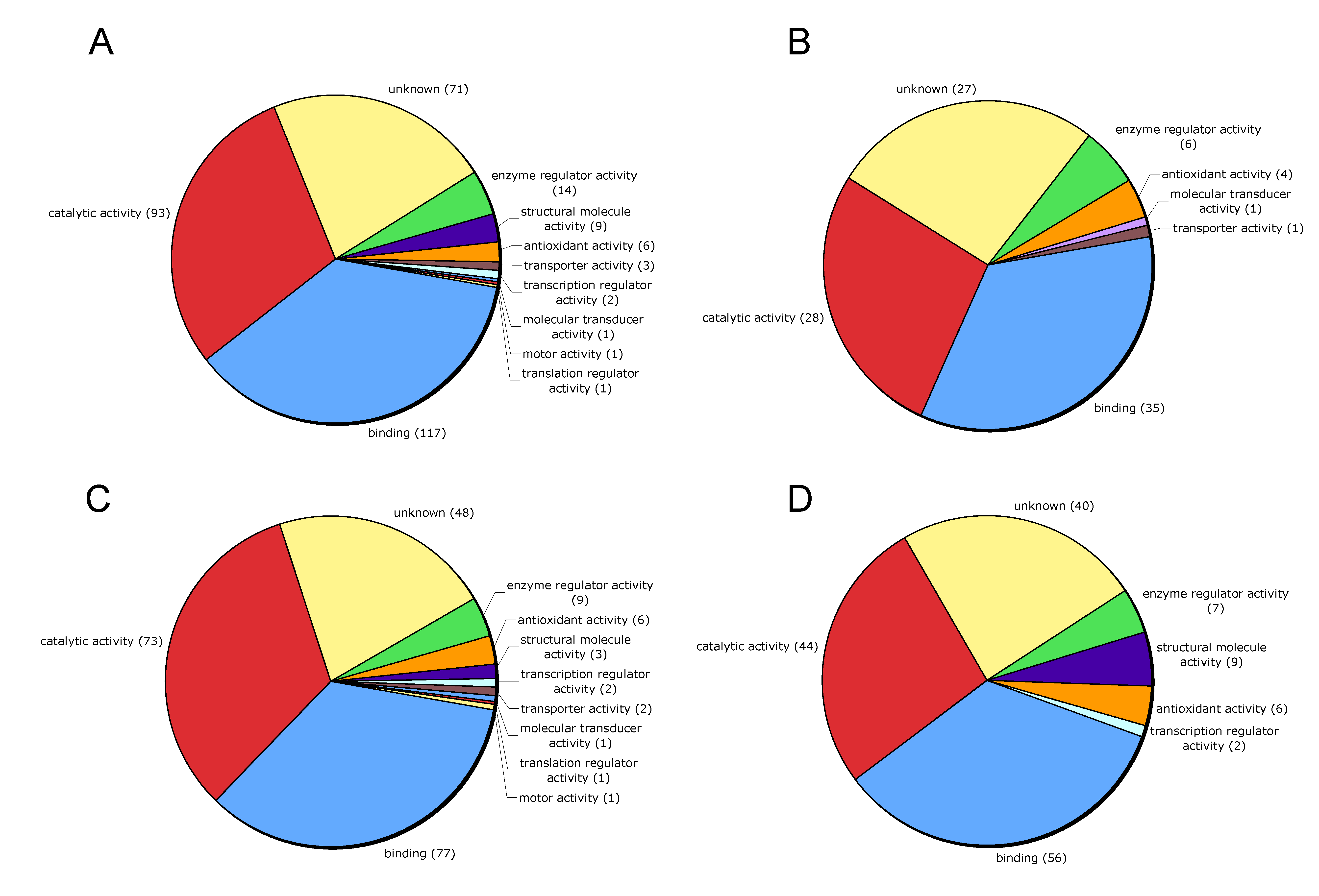

Supplement: Figure S1 — Distribution of Molecular Function Gene Ontology terms (level 2) compared between stages/genders of B. malayi. A. total set of ESP identified, B. ESP identified in microfilariae, C. ESP identified in females, D. ESP identified in males. (1.86 MB TIF) [file pntd.0000326.s001.tif]

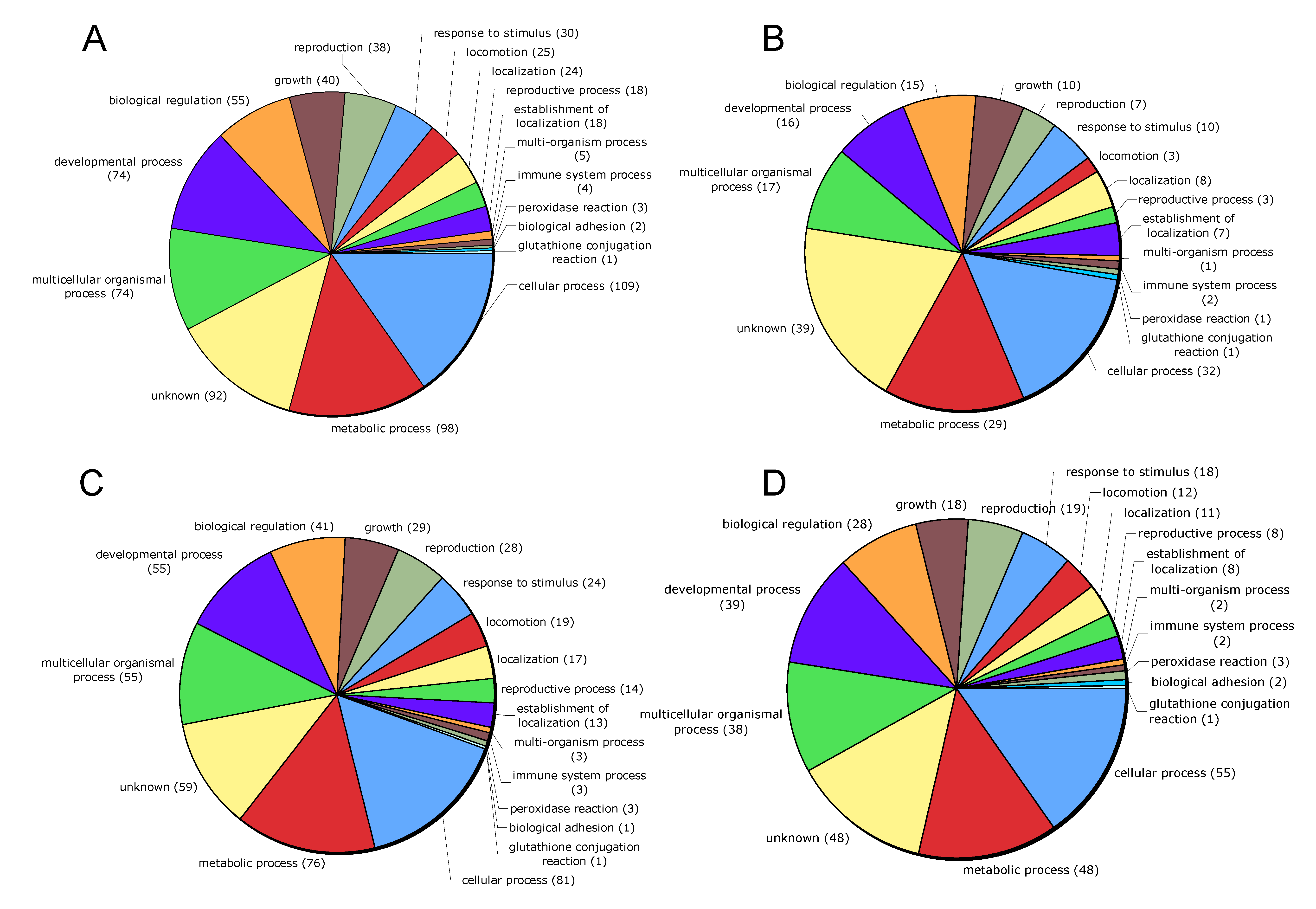

Supplement: Figure S2 — Distribution of Biological Process Gene Ontology terms (level 2) compared between stages/genders of B. malayi. A. total set of ESP identified, B. ESP identified in microfilariae, C. ESP identified in females, D. ESP identified in males. (2.39 MB TIF) [file pntd.0000326.s002.tif]
