# Supplementary material for: Stage- and Gender-Specific Proteomic Analysis of Brugia malayi Excretory-Secretory Products
Source: PLoS Negl Trop Dis. 2008 Oct 29;2(10):e326. doi: 10.1371/journal.pntd.0000326 (PMC2569413; doi:10.1371/journal.pntd.0000326)
Supplement: Table S1 — Proteins identified in ESP from microfilaria, female and male B. malayi. TP: Prediction of subcellular localization by TargetP, (M): Mitochondrion, (S): Secretory pathway (−): Any other location. RC: Reliability class, from 1 to 5, where 1 indicates the strongest prediction. GO: Gene Ontology. EC: Enzyme Commission number. NQPCT: Prorated Query Count Percentage values. (0.78 MB DOC) [file pntd.0000326.s003.doc]

| **Proteins Found in Microfilariae, Female and Male worms** | | | | | | | | | | | | | | |
| --- | --- | --- | --- | --- | --- | --- | --- | --- | --- | --- | --- | --- | --- | --- |
|  | **Uniprot ID** | **Pub_locus** | **TIGR locus** | **DESCRIPTION** | **TP** | **RC** | **SP** | **SecP** | **#GOs** | **GO IDs** | **Enzyme** | **NQPCT** | | |
|  |  |  |  |  |  |  |  |  |  |  |  | **Mf** | **Fw** | **Mw** |
| 1 | A8PKM4 | Bm1_29130 | 14940.m00172 | A8PKM4_BRUMA Triosephosphate isomerase, putative | - | 3 | N | 0.36 | 10 | P:GO:0006094 P:GO:0006633 F:GO:0004807 P:GO:0006098 P:GO:0006096 P:GO:0006000 P:GO:0006013 P:GO:0006020 P:GO:0015976 P:GO:0046486 | EC:5.3.1.1, | 3.48 | 16.87 | 3.73 |
| 2 | A8QEB1 | Bm1_51000 | 14992.m10974 | A8QEB1_BRUMA Putative uncharacterized protein | - | 2 | N | 0.51 | 4 | F:GO:0008289 F:GO:0019841 P:GO:0019538 F:GO:0005515 | - | 4.20 | 5.19 | 5.55 |
| 3 | A8NW22 | Bm1_11105 | 13673.m00035 | A8NW22_BRUMA Recombinant antigen R1, identical | S | 1 | Y | 0.70 | 0 |  | - | 14.35 | 0.27 | 0.15 |
| 4 | P67877 | Bm1_40465 | 14972.m07803 | GPXC_BRUMA Cuticular glutathione peroxidase precursor | S | 1 | Y | 0.59 | 5 | F:GO:0004602 C:GO:0005576 P:GO:0006979 P:GO:0006749 P:GO:0006804 | EC:1.11.1.9, | 0.29 | 2.74 | 7.69 |
| 5 | A8QH34 | Bm1_56305 | 15373.m00009 | A8QH34_BRUMA Leucyl aminopeptidase, putative | S | 1 | Y | 0.86 | 6 | F:GO:0004180 F:GO:0004177 C:GO:0005576 P:GO:0006508 F:GO:0008237 P:GO:0042246 | EC:3.4.11, | 0.58 | 7.72 | 1.83 |
| 6 | A8PJU3 | Bm1_28435 | 14930.m00337 | A8PJU3_BRUMA Bm-MIF-1, identical | - | 2 | N | 0.51 | 7 | P:GO:0001516 P:GO:0043030 P:GO:0007166 F:GO:0005125 C:GO:0005615 P:GO:0008283 P:GO:0043066 |  | 0.72 | 8.91 | 0.15 |
| 7 | A8PGM6 | Bm1_24940 | 14731.m01012 | A8PGM6_BRUMA Galectin, putative | - | 1 | N | 0.35 | 2 | F:GO:0005515 F:GO:0005529 |  | 2.61 | 5.22 | 0.56 |
| 8 | A8PFE3 | Bm1_24115 | 14703.m00079 | A8PFE3_BRUMA Enolase, putative | - | 4 | N | 0.53 | 9 | C:GO:0000015 C:GO:0005811 F:GO:0004634 F:GO:0000287 P:GO:0006096 P:GO:0000162 P:GO:0006094 P:GO:0006571 P:GO:0009094 | EC:4.2.1.11, | 1.16 | 2.54 | 4.69 |
| 9 | A8Q2C4 | Bm1_41005 | 14973.m02599 | A8Q2C4_BRUMA OV-16 antigen, putative | - | 2 | N | 0.60 | 0 |  | - | 4.64 | 0.75 | 1.83 |
| 10 | O97392 | Bm1_09950 | 13531.m00015 | O97392_BRUMA Gamma-glutamyl transpeptidase precursor | S | 1 | Y | 0.69 | 5 | F:GO:0003840 P:GO:0006691 P:GO:0006693 P:GO:0006749 P:GO:0019530 | EC:2.3.2.2, | 2.75 | 3.35 | 0.81 |
| 11 | A8NQM6 | Bm1_07780 | 13333.m00082 | A8NQM6_BRUMA Immunogenic protein 3, putative | S | 1 | Y | 0.88 | 0 |  | - | 3.91 | 0.98 | 1.32 |
| 12 | O16159 | Bm1_56600 | 15418.m00009 | O16159_BRUMA Cystatin-type cysteine proteinase inhibitor | S | 1 | Y | 0.96 | 1 | F:GO:0004869 |  | 1.74 | 1.83 | 1.39 |
| 13 | Q9BJC9 |  | - | Q9BJC9_BRUMA Major allergen | S | 2 | Y | 0.76 | 5 | P:GO:0018996 P:GO:0010171 P:GO:0040010 P:GO:0002119 P:GO:0040007 | - | 2.03 | 1.69 | 1.02 |
| 14 | Q6T8C4 |  | - | Q6T8C4_BRUMA Superoxide dismutase [Cu-Zn] | - | 2 | N | 0.39 | 12 | C:GO:0005737 P:GO:0001306 F:GO:0016209 P:GO:0040028 F:GO:0005507 P:GO:0019430 F:GO:0004784 P:GO:0040010 C:GO:0005576 F:GO:0008270 P:GO:0006801 F:GO:0046872 | EC:1.15.1.1, | 1.74 | 0.58 | 1.17 |
| 15 | Q4VWF8 | Bm1_04665 | 13047.m00009 | Q4VWF8_BRUMA Independent phosphoglycerate mutase isoform 1 | - | 2 | N | 0.49 | 14 | P:GO:0040010 P:GO:0018991 P:GO:0009792 F:GO:0030145 P:GO:0010171 P:GO:0002119 F:GO:0004619 P:GO:0040011 C:GO:0005737 P:GO:0008340 F:GO:0003824 F:GO:0046872 P:GO:0006094 P:GO:0006096 | EC:5.4.2.1, | 0.29 | 0.58 | 2.42 |
| 16 | A8QGU4 | Bm1_55850 | 15309.m00029 | A8QGU4_BRUMA Cyclophilin-type peptidyl-prolyl cis-trans isomerase-2, Bmcyp-2 | - | 3 | N | 0.32 | 3 | P:GO:0006457 F:GO:0003755 P:GO:0009792 | EC:5.2.1.8, | 1.45 | 0.47 | 1.10 |
| 17 | A8NVU3 | Bm1_10970 | 13662.m00125 | A8NVU3_BRUMA 14-3-3-like protein 2, putative | - | 1 | N | 0.33 | 28 | P:GO:0007265 P:GO:0001751 P:GO:0008103 P:GO:0045167 P:GO:0009792 P:GO:0035046 P:GO:0008355 P:GO:0040010 P:GO:0010070 P:GO:0008283 P:GO:0000132 C:GO:0045172 F:GO:0019904 P:GO:0002009 P:GO:0009949 P:GO:0007059 P:GO:0002119 F:GO:0004863 P:GO:0045448 F:GO:001 |  | 0.72 | 0.30 | 1.41 |
| 18 | A8P5I2 | Bm1_16955 | 14271.m00285 | A8P5I2_BRUMA Inorganic pyrophosphatase, putative | S | 1 | Y | 0.52 | 8 | C:GO:0005737 P:GO:0002119 P:GO:0040010 F:GO:0000287 P:GO:0000003 P:GO:0009792 F:GO:0004427 P:GO:0006119 | EC:3.6.1.1, | 1.01 | 0.41 | 0.66 |
| 19 | A8P9K9 | Bm1_19875 | 14469.m00102 | A8P9K9_BRUMA Putative uncharacterized protein | - | 1 | N | 0.34 | 0 |  | - | 0.58 | 0.24 | 1.24 |
| 20 | A8Q921 | Bm1_46705 | 14981.m02380 | A8Q921_BRUMA Thioredoxin, identical | - | 1 | N | 0.65 | 5 | P:GO:0045454 F:GO:0009055 F:GO:0016209 F:GO:0005515 P:GO:0006118 |  | 0.58 | 0.71 | 0.66 |
| 21 | A8NQI4 | Bm1_07685 | 13325.m00230 | A8NQI4_BRUMA DJ-1 family protein | - | 3 | N | 0.41 | 13 | P:GO:0007265 F:GO:0003723 P:GO:0008344 C:GO:0005739 P:GO:0051899 P:GO:0060081 P:GO:0051583 F:GO:0005515 P:GO:0042743 P:GO:0008283 F:GO:0051920 P:GO:0042542 C:GO:0005634 | EC:1.11.1.15, | 0.87 | 0.37 | 0.59 |
| 22 | A8PZA5 | Bm1_38150 | 14972.m07327 | A8PZA5_BRUMA Putative uncharacterized protein | S | 1 | Y | 0.62 | 0 |  | - | 0.72 | 0.34 | 0.66 |
| 23 | A8P382 | Bm1_15250 | 14164.m00122 | A8P382_BRUMA Transthyretin-like family protein | S | 2 | N | 0.90 | 0 |  | - | 0.29 | 1.02 | 0.37 |
| 24 | A8PII4 | Bm1_26590 | 14830.m00078 | A8PII4_BRUMA Hypothetical UPF0205 protein C40H1.5 in chromosome III, putative | S | 1 | Y | 0.89 | 0 |  | - | 0.29 | 0.85 | 0.37 |
| 25 | Q6YNZ2 | Bm1_24035 | 14702.m00390 | Q6YNZ2_BRUMA Peptidyl-prolyl cis-trans isomerase | S | 4 | N | 0.51 | 7 | C:GO:0005615 P:GO:0006457 C:GO:0005788 C:GO:0042470 P:GO:0051169 F:GO:0042277 F:GO:0003755 | EC:5.2.1.8, | 0.87 | 0.17 | 0.29 |
| 26 | Q17198 | Bm1_42870 | 14975.m04515 | Q17198_BRUMA Brugia malayi antigen | - | 2 | Y | 0.87 | 0 |  | - | 0.72 | 0.20 | 0.15 |
| 27 | A8Q729 | Bm1_44840 | 14979.m04477 | A8Q729_BRUMA Glutathione S-transferase, N-terminal domain containing protein | S | 1 | Y | 0.76 | 4 | F:GO:0004364 C:GO:0005737 P:GO:0006749 P:GO:0006803 | - | 0.29 | 0.54 | 0.07 |
| 28 | A8NNQ3 | Bm1_06445 | 13250.m00031 | A8NNQ3_BRUMA Transthyretin-like family protein | - | 2 | N | 0.84 | 0 |  | - | 0.29 | 0.20 | 0.37 |
| 29 | A8NKK9 | Bm1_04380 | 12984.m00011 | A8NKK9_BRUMA Transthyretin-like family protein | S | 2 | Y | 0.96 | 0 |  | - | 0.14 | 0.17 | 0.51 |
| 30 | A8PJQ3 | Bm1_28235 | 14927.m00101 | A8PJQ3_BRUMA Cytochrome c type-1, putative | - | 1 | N | 0.75 | 7 | F:GO:0009055 F:GO:0005506 C:GO:0005759 F:GO:0020037 P:GO:0006118 P:GO:0006810 C:GO:0005746 |  | 0.29 | 0.20 | 0.22 |
| 31 | A8Q4E4 | Bm1_42885 | 14975.m04518 | A8Q4E4_BRUMA Filarial antigen Av33, putative | S | 1 | Y | 0.68 | 2 | C:GO:0005576 F:GO:0019828 |  | 0.14 | 0.14 | 0.22 |
| 32 | A8Q5Y3 | Bm1_43635 | 14977.m04975 | A8Q5Y3_BRUMA Hypothetical UPF0205 protein T07C12.7 in chromosome V, putative | S | 3 | Y | 0.95 | 0 |  | - | 0.14 | 0.03 | 0.15 |
|  |  |  |  |  |  |  |  |  |  |  |  |  |  |  |
| **Proteins Found only in Adults Stages** | | | | | | | | | | | | | | |
|  | **Uniprot ID** | **Pub_locus** | **TIGR locus** | **DESCRIPTION** | **TP** | **RC** | **SP** | **SecP** | **#GOs** | **GO IDs** | **Enzyme** | **NQPCT** | | |
|  |  |  |  |  |  |  |  |  |  |  |  | **Mf** | **Fw** | **Mw** |
| 33 | A8QFI4 | Bm1_53510 | 15059.m00091 | A8QFI4_BRUMA Myotactin form B, putative | - | 4 | N | 0.35 | 1 | P:GO:0007275 |  | 0.00 | 5.24 | 7.17 |
| 34 | A8P3E5 | Bm1_15350 | 14176.m00093 | A8P3E5_BRUMA Fructose-bisphosphate aldolase 1, putative | - | 1 | N | 0.40 | 13 | F:GO:0042802 P:GO:0006000 P:GO:0043627 P:GO:0035094 P:GO:0006941 P:GO:0006096 F:GO:0004332 P:GO:0009408 P:GO:0006013 P:GO:0006020 P:GO:0006094 P:GO:0006098 P:GO:0015976 | EC:4.1.2.13, | 0.00 | 1.29 | 6.00 |
| 35 | A8NPW6 | Bm1_07275 | 13311.m00333 | A8NPW6_BRUMA Core-2/I-Branching enzyme family protein | - | 1 | N | 0.67 | 2 | F:GO:0008375 C:GO:0016020 | - | 0.00 | 0.10 | 5.68 |
| 36 | A8P0Q6 | Bm1_13605 | 14015.m00091 | A8P0Q6_BRUMA Major Sperm Protein | - | 2 | N | 0.62 | 5 | C:GO:0005856 C:GO:0005737 P:GO:0000003 F:GO:0005198 P:GO:0009792 |  | 0.00 | 0.38 | 4.63 |
| 37 | A8NLB0 | Bm1_04870 | 13066.m00251 | A8NLB0_BRUMA Putative uncharacterized protein | - | 2 | N | 0.78 | 2 | F:GO:0008375 C:GO:0016020 | - | 0.00 | 0.10 | 3.18 |
| 38 | A8NZQ9 | Bm1_12945 | 13929.m00009 | A8NZQ9_BRUMA Lethal protein 805, isoform d, putative | S | 3 | Y | 0.60 | 8 | P:GO:0009792 P:GO:0040011 C:GO:0044464 P:GO:0018996 P:GO:0007154 P:GO:0008340 P:GO:0002119 P:GO:0048523 |  | 0.00 | 0.37 | 2.53 |
| 39 | A9XG48 | Bm1_50995 | 14992.m10973 | A9XG48_BRUMA L3R15 repetitive antigen | - | 1 | N | 0.63 | 1 | F:GO:0005488 |  | 0.00 | 1.04 | 1.85 |
| 40 | A8Q119 | Bm1_40580 | 14972.m07829 | A8Q119_BRUMA Glycosyl hydrolases family 31 protein | S | 5 | N | 0.59 | 2 | F:GO:0015926 P:GO:0005975 |  | 0.00 | 1.49 | 1.39 |
| 41 | A8Q5Z6 | Bm1_43675 | 14977.m04983 | A8Q5Z6_BRUMA Heat shock 70 kDa protein, putative | S | 4 | N | 0.28 | 10 | F:GO:0005524 P:GO:0000003 P:GO:0040007 P:GO:0010171 P:GO:0009792 F:GO:0005515 P:GO:0002119 P:GO:0009408 P:GO:0040011 P:GO:0008340 |  | 0.00 | 1.05 | 0.95 |
| 42 | A8P0Q4 | Bm1_13600 | 14015.m00090 | A8P0Q4_BRUMA Major sperm protein 2 , putative cytoskeletal MSP | - | 2 | N | 0.75 | 3 | C:GO:0005856 F:GO:0005198 C:GO:0005737 |  | 0.00 | 0.13 | 1.74 |
| 43 | A8Q0F4 | Bm1_40185 | 14972.m07743 | A8Q0F4_BRUMA Calsequestrin family protein | S | 2 | Y | 0.76 | 3 | C:GO:0033018 C:GO:0005739 F:GO:0005509 |  | 0.00 | 1.32 | 0.22 |
| 44 | A8QEM1 | Bm1_51495 | 14992.m11078 | A8QEM1_BRUMA Heat shock protein 90, putative | - | 2 | N | 0.18 | 16 | P:GO:0006952 C:GO:0005829 P:GO:0042026 P:GO:0045585 P:GO:0007049 F:GO:0030235 P:GO:0040024 P:GO:0000003 P:GO:0009408 F:GO:0005524 C:GO:0048471 F:GO:0051082 P:GO:0006986 P:GO:0045429 F:GO:0042803 P:GO:0008340 |  | 0.00 | 1.08 | 0.44 |
| 45 | A8Q612 | Bm1_43730 | 14977.m04994 | A8Q612_BRUMA Lactate dehydrogenase., putative | M | 5 | N | 0.53 | 7 | C:GO:0005737 F:GO:0005488 P:GO:0019642 F:GO:0004459 P:GO:0006100 P:GO:0006094 P:GO:0006534 | EC:1.1.1.27, | 0.00 | 0.95 | 0.44 |
| 46 | A8NU08 | Bm1_09810 | 13507.m00011 | A8NU08_BRUMA Lethal protein 805, isoform d, putative | - | 1 | N | 0.52 | 10 | P:GO:0009792 P:GO:0040011 C:GO:0044464 F:GO:0016787 P:GO:0018996 P:GO:0007167 P:GO:0008340 P:GO:0002119 P:GO:0048523 P:GO:0050793 |  | 0.00 | 0.61 | 0.76 |
| 47 | A8Q940 | Bm1_46740 | 14981.m02387 | A8Q940_BRUMA Galactoside-binding lectin family protein | S | 2 | Y | 0.67 | 2 | F:GO:0005515 F:GO:0005529 |  | 0.00 | 0.10 | 1.24 |
| 48 | A8Q2M4 | Bm1_41425 | 14973.m02677 | A8Q2M4_BRUMA BM20, putative | S | 1 | Y | 0.95 | 4 | C:GO:0005576 F:GO:0005504 F:GO:0019841 F:GO:0016918 |  | 0.00 | 0.54 | 0.66 |
| 49 | A8PE64 | Bm1_23190 | 14656.m00228 | A8PE64_BRUMA Hypothetical 86.9 kDa protein C30C11.4 in chromosome III, putative | - | 2 | N | 0.26 | 1 | F:GO:0005524 | - | 0.00 | 1.05 | 0.15 |
| 50 | A8PJI9 | Bm1_27905 | 14920.m00410 | A8PJI9_BRUMA Polyubiquitin, putative | M | 4 | N | 0.51 | 8 | P:GO:0002119 P:GO:0045941 F:GO:0030528 P:GO:0016567 P:GO:0000003 P:GO:0009792 P:GO:0040007 C:GO:0005634 |  | 0.00 | 0.98 | 0.07 |
| 51 | A8PJM3 | Bm1_28070 | 14922.m00060 | A8PJM3_BRUMA Oxidoreductase, aldo/keto reductase family protein | - | 4 | N | 0.38 | 12 | P:GO:0042840 C:GO:0016324 P:GO:0046185 P:GO:0019853 F:GO:0047939 F:GO:0004032 C:GO:0005829 P:GO:0006000 P:GO:0006012 P:GO:0006013 P:GO:0006090 P:GO:0046486 | EC:1.1.1.19, EC:1.1.1.21, | 0.00 | 0.54 | 0.51 |
| 52 | A8Q5I4 | Bm1_43130 | 14977.m04868 | A8Q5I4_BRUMA Serine carboxypeptidase F41C3.5, putative | S | 2 | Y | 0.89 | 4 | C:GO:0005739 F:GO:0005515 F:GO:0004185 P:GO:0006508 |  | 0.00 | 0.17 | 0.81 |
| 53 | A8QGM5 | Bm1_55510 | 15269.m00009 | A8QGM5_BRUMA Myotactin form A, putative | - | 1 | N | 0.56 | 1 | P:GO:0007275 |  | 0.00 | 0.59 | 0.37 |
| 54 | A8PVX0 | Bm1_35885 | 14971.m02817 | A8PVX0_BRUMA MFP2, putative | - | 1 | N | 0.42 | 0 |  | - | 0.00 | 0.03 | 0.88 |
| 55 | A8PII3 | Bm1_26585 | 14830.m00077 | A8PII3_BRUMA Hypothetical UPF0205 protein C40H1.5 in chromosome III, putative | S | 1 | Y | 0.52 | 0 |  | - | 0.00 | 0.75 | 0.07 |
| 56 | A8Q0Z9 | Bm1_40520 | 14972.m07815 | A8Q0Z9_BRUMA Lectin C-type domain containing protein | - | 2 | N | 0.69 | 1 | F:GO:0005488 | - | 0.00 | 0.54 | 0.22 |
| 57 | A8Q3K1 | Bm1_41940 | 14975.m04553 | A8Q3K1_BRUMA Glyceraldehyde 3-phosphate dehydrogenase, putative | M | 5 | N | 0.54 | 5 | F:GO:0051287 P:GO:0006096 F:GO:0004365 C:GO:0005739 P:GO:0006094 | EC:1.2.1.12, | 0.00 | 0.30 | 0.44 |
| 58 | Q8MY58 | Bm1_12055 | 13806.m00014 | Q8MY58_BRUMA Aspartic protease BmAsp-2 | S | 1 | Y | 0.69 | 5 | C:GO:0005764 P:GO:0012503 F:GO:0004194 P:GO:0006508 F:GO:0004190 | EC:3.4.23.1, | 0.00 | 0.30 | 0.44 |
| 59 | A8QAU0 | Bm1_47625 | 14990.m07644 | A8QAU0_BRUMA Glucose phosphate isomerase a, putative | S | 1 | Y | 0.74 | 9 | C:GO:0005737 P:GO:0006094 F:GO:0004347 F:GO:0005515 P:GO:0009792 P:GO:0006096 P:GO:0005982 P:GO:0005985 P:GO:0006098 | EC:5.3.1.9, | 0.00 | 0.51 | 0.22 |
| 60 | P48817 | Bm1_50135 | 14990.m08156 | NDK_BRUMA Nucleoside diphosphate kinase | - | 2 | N | 0.33 | 33 | P:GO:0009792 C:GO:0030027 P:GO:0007155 F:GO:0004550 P:GO:0006241 P:GO:0046777 P:GO:0045682 P:GO:0006355 C:GO:0005880 F:GO:0005525 P:GO:0045786 P:GO:0008285 F:GO:0008017 P:GO:0006165 C:GO:0001726 P:GO:0018105 P:GO:0007067 P:GO:0045618 F:GO:0003700 P:GO:000 | EC:2.7.4.6, | 0.00 | 0.14 | 0.51 |
| 61 | A8P3D2 | Bm1_15330 | 14174.m00063 | A8P3D2_BRUMA Glycosyl hydrolase family 20, catalytic domain containing protein | S | 2 | Y | 0.59 | 17 | P:GO:0007040 P:GO:0042552 C:GO:0005764 F:GO:0004563 P:GO:0019915 P:GO:0050885 P:GO:0019953 P:GO:0006689 P:GO:0050884 P:GO:0007605 P:GO:0007628 P:GO:0001501 C:GO:0016020 F:GO:0043169 P:GO:0001575 P:GO:0006027 P:GO:0006040 | EC:3.2.1.52, | 0.00 | 0.14 | 0.44 |
| 62 | A8P102 | Bm1_13815 | 14037.m00201 | A8P102_BRUMA Prolyl oligopeptidase family protein | - | 1 | N | 0.47 | 3 | C:GO:0005634 F:GO:0004287 P:GO:0006508 | EC:3.4.21.26, | 0.00 | 0.37 | 0.15 |
| 63 | Q9BMK6 |  | - | Q9BMK6_BRUMA Thioredoxin peroxidase | - | 3 | N | 0.51 | 7 | F:GO:0008379 C:GO:0005737 P:GO:0040018 P:GO:0006979 F:GO:0016209 F:GO:0016491 P:GO:0006804 |  | 0.00 | 0.36 | 0.15 |
| 64 | A8P795 | Bm1_18190 | 14355.m00213 | A8P795_BRUMA 14-3-3-like protein 2, putative | - | 1 | N | 0.39 | 25 | P:GO:0007265 P:GO:0001751 P:GO:0008103 P:GO:0008355 P:GO:0040010 P:GO:0008283 C:GO:0042470 C:GO:0045172 F:GO:0019904 P:GO:0007059 P:GO:0042493 C:GO:0014069 F:GO:0004863 P:GO:0045448 F:GO:0016483 F:GO:0032403 F:GO:0008134 C:GO:0005625 P:GO:0006588 P:GO:000 |  | 0.00 | 0.30 | 0.20 |
| 65 | A8Q946 | Bm1_46750 | 14981.m02389 | A8Q946_BRUMA Galectin, putative | - | 2 | N | 0.39 | 2 | F:GO:0005515 F:GO:0005529 |  | 0.00 | 0.40 | 0.09 |
| 66 | A8Q7J8 | Bm1_45475 | 14979.m04613 | A8Q7J8_BRUMA Immunoglobulin I-set domain containing protein |  |  | N |  | 2 | F:GO:0005509 F:GO:0005507 | - | 0.00 | 0.17 | 0.29 |
| 67 | A8QBZ7 | Bm1_49010 | 14990.m07926 | A8QBZ7_BRUMA FKBP-type peptidyl-prolyl cis-trans isomerase-12, BmFKBP-12 | - | 1 | N | 0.56 | 5 | C:GO:0005737 P:GO:0006457 F:GO:0016887 F:GO:0005524 F:GO:0003755 | EC:5.2.1.8, | 0.00 | 0.24 | 0.22 |
| 68 | A8NU55 | Bm1_09895 | 13522.m00031 | A8NU55_BRUMA Basement membrane proteoglycan, putative | - | 3 | N | 0.48 | 2 | P:GO:0009987 P:GO:0048731 |  | 0.00 | 0.03 | 0.37 |
| 69 | A8NN00 | Bm1_05930 | 13207.m00046 | A8NN00_BRUMA Muscle positioning protein 4, putative | - | 1 | N | 0.60 | - | - | - | 0.00 | 0.17 | 0.22 |
| 70 | A8P9U1 | Bm1_20065 | 14486.m00069 | A8P9U1_BRUMA Transthyretin-like family protein | S | 2 | Y | 0.62 | 0 |  | - | 0.00 | 0.03 | 0.29 |
| 71 | Q17172 |  | - | TDX2_BRUMA Thioredoxin peroxidase 2 | - | 3 | N | 0.57 | 7 | F:GO:0008379 C:GO:0005737 P:GO:0040018 P:GO:0006979 F:GO:0016209 F:GO:0016491 P:GO:0006804 |  | 0.00 | 0.15 | 0.15 |
| 72 | A8QFI5 | Bm1_53515 | 15059.m00092 | A8QFI5_BRUMA Putative uncharacterized protein | - | 5 | N | 0.32 | 6 | P:GO:0018996 P:GO:0009792 F:GO:0005515 P:GO:0002119 P:GO:0040011 P:GO:0008340 |  | 0.00 | 0.14 | 0.15 |
| 73 | A8QCR3 | Bm1_49920 | 14990.m08110 | A8QCR3_BRUMA Moesin/ezrin/radixin homolog 1, putative | - | 2 | N | 0.73 | 25 | C:GO:0030175 P:GO:0045176 P:GO:0040010 C:GO:0019898 C:GO:0032420 C:GO:0001726 P:GO:0035148 P:GO:0009792 P:GO:0007015 P:GO:0051016 C:GO:0031974 P:GO:0030033 P:GO:0010171 C:GO:0001931 C:GO:0005938 P:GO:0002119 F:GO:0003779 C:GO:0031941 P:GO:0040011 C:GO:001 |  | 0.00 | 0.20 | 0.07 |
| 74 | A8PEL0 | Bm1_23560 | 14677.m00169 | A8PEL0_BRUMA Calreticulin, putative | S | 1 | Y | 0.05 | 13 | P:GO:0043071 F:GO:0008270 C:GO:0009897 F:GO:0005509 P:GO:0006457 P:GO:0030866 C:GO:0005615 C:GO:0005792 C:GO:0005788 P:GO:0040011 F:GO:0051082 F:GO:0005529 P:GO:0040020 |  | 0.00 | 0.03 | 0.22 |
| 75 | A8P872 | Bm1_18805 | 14387.m00341 | A8P872_BRUMA Papain family cysteine protease containing protein | S | 1 | N | 0.54 | 2 | P:GO:0006508 F:GO:0004197 | EC:3.4.22, | 0.00 | 0.10 | 0.15 |
| 76 | A8QHK3 | Bm1_57145 | 15487.m00008 | A8QHK3_BRUMA Fibronectin type III domain containing protein | - | 1 | N | 0.64 | 5 | P:GO:0018996 P:GO:0009792 P:GO:0002119 P:GO:0040011 P:GO:0008340 |  | 0.00 | 0.10 | 0.15 |
| 77 | A8PC30 | Bm1_21620 | 14590.m00346 | A8PC30_BRUMA Profilin family protein | - | 3 | N | 0.63 | 9 | P:GO:0002165 P:GO:0007417 P:GO:0000910 P:GO:0030036 P:GO:0048513 P:GO:0009653 P:GO:0048477 F:GO:0003779 C:GO:0015629 |  | 0.00 | 0.17 | 0.07 |
| 78 | A8P4L2 | Bm1_16260 | 14237.m00401 | A8P4L2_BRUMA Proteasome subunit alpha type 3, putative | - | 2 | N | 0.43 | 5 | F:GO:0005515 C:GO:0005634 F:GO:0004298 C:GO:0005839 P:GO:0006511 |  | 0.00 | 0.07 | 0.15 |
| 79 | A8NUI5 | Bm1_10120 | 13555.m00075 | A8NUI5_BRUMA Viral A-type inclusion protein repeat containing protein | - | 2 | N | 0.23 | 7 | P:GO:0006366 C:GO:0005737 C:GO:0016020 C:GO:0005794 P:GO:0019438 F:GO:0003713 F:GO:0005515 | - | 0.00 | 0.03 | 0.15 |
| 80 | A8P138 | Bm1_13900 | 14039.m00116 | A8P138_BRUMA Putative uncharacterized protein | S | 1 | Y | 0.79 | 0 |  | - | 0.00 | 0.03 | 0.15 |
| 81 | A8QH67 | Bm1_56470 | 15397.m00021 | A8QH67_BRUMA Chaperonin-10 kDa, putative | - | 5 | N | 0.21 | 9 | P:GO:0009792 P:GO:0006457 F:GO:0051087 P:GO:0007565 P:GO:0006919 F:GO:0005524 C:GO:0005759 F:GO:0051082 P:GO:0006986 |  | 0.00 | 0.03 | 0.15 |
| 82 | Q9NAS2 |  | - | Q9NAS2_BRUMA Macrophage migration inhibitory factor 2 | - | 4 | N | 0.71 | 2 | F:GO:0003674 P:GO:0008150 | - | 0.00 | 0.03 | 0.15 |
| 83 | A8Q227 | Bm1_37185 | 14972.m07130 | A8Q227_BRUMA Saposin-like type B, region 1 family protein | S | 1 | Y | 0.71 | 1 | P:GO:0006629 | - | 0.00 | 0.07 | 0.07 |
| 84 | A8NH41 | Bm1_02365 | 12681.m00095 | A8NH41_BRUMA Putative uncharacterized protein | - | 2 | N | 0.65 | 12 | F:GO:0008270 P:GO:0019370 P:GO:0006954 F:GO:0004463 F:GO:0005515 F:GO:0004301 F:GO:0004179 P:GO:0006508 P:GO:0006805 P:GO:0009636 P:GO:0006749 C:GO:0016020 | EC:3.3.2.6, EC:3.3.2.10, | 0.00 | 0.03 | 0.07 |
| 85 | A8NK26 | Bm1_04195 | 12949.m00009 | A8NK26_BRUMA Transaldolase, putative | - | 2 | N | 0.59 | 3 | C:GO:0005737 F:GO:0004801 P:GO:0006098 | EC:2.2.1.2, | 0.00 | 0.03 | 0.07 |
| 86 | A8Q4C0 | Bm1_42810 | 14975.m04503 | A8Q4C0_BRUMA MTAP, putative | - | 1 | N | 0.38 | 3 | P:GO:0006139 F:GO:0017061 F:GO:0004645 | EC:2.4.2.28, EC:2.4.1.1, | 0.00 | 0.03 | 0.07 |
|  |  |  |  |  |  |  |  |  |  |  |  |  |  |  |
| **Proteins found in Microfilariae and Female Worms** | | | | | | | | | | | | | | |
|  | **Uniprot ID** | **Pub_locus** | **TIGR locus** | **DESCRIPTION** | **TP** | **RC** | **SP** | **SecP** | **#GOs** | **GO IDs** | **Enzyme** | **NQPCT** | | |
|  |  |  |  |  |  |  |  |  |  |  |  | **Mf** | **Fw** | **Mw** |
| 87 | A8NVF8 | Bm1_10700 | 13632.m00191 | A8NVF8_BRUMA OV39 antigen-like | - | 2 | N | 0.85 | 0 |  | - | 0.43 | 0.20 | 0.00 |
| 88 | A8P2T1 | Bm1_14965 | 14122.m00164 | A8P2T1_BRUMA Putative uncharacterized protein | S | 1 | Y | 0.82 | 4 | P:GO:0007275 F:GO:0004872 C:GO:0016021 P:GO:0007165 |  | 0.29 | 0.07 | 0.00 |
| 89 | A8QCR6 | Bm1_49930 | 14990.m08112 | A8QCR6_BRUMA G15-6A protein, putative | S | 2 | Y | 0.81 | 0 |  | - | 0.29 | 0.07 | 0.00 |
| 90 | A8QDX2 | Bm1_50415 | 14992.m10856 | A8QDX2_BRUMA Calmodulin, putative | - | 1 | N | 0.70 | 11 | C:GO:0016028 P:GO:0016060 F:GO:0005509 C:GO:0000922 P:GO:0007052 P:GO:0005513 P:GO:0051383 F:GO:0032036 P:GO:0016062 P:GO:0016061 P:GO:0006468 |  | 0.29 | 0.03 | 0.00 |
| 91 | A8QBA4 | Bm1_51000 | 14990.m07743 | A8QBA4_BRUMA Phosphatidylinositol 3-and 4-kinase family protein | - | 1 | N | 0.38 | 10 | P:GO:0018105 F:GO:0004674 P:GO:0046854 F:GO:0005515 F:GO:0004428 P:GO:0000184 P:GO:0046777 C:GO:0005737 C:GO:0005634 P:GO:0009069 | EC:2.7.11, | 0.14 | 0.10 | 0.00 |
| 92 | A8P0S4 | Bm1_13650 | 14026.m00009 | A8P0S4_BRUMA Hypothetical UPF0205 protein C40H1.5 in chromosome III, putative | S | 1 | Y | 0.50 | 0 |  | - | 0.14 | 0.07 | 0.00 |
| 93 | A8QE26 | Bm1_50630 | 14992.m10900 | A8QE26_BRUMA Putative uncharacterized protein | - | 1 | N | 0.74 | 3 | P:GO:0002009 P:GO:0018996 P:GO:0040010 |  | 0.14 | 0.03 | 0.00 |
|  |  |  |  |  |  |  |  |  |  |  |  |  |  |  |
| **Proteins Foun in Microfilariae and Male worms** | | | | | | | | | | | | | | |
|  | **Uniprot ID** | **Pub_locus** | **TIGR locus** | **DESCRIPTION** | **TP** | **RC** | **SP** | **SecP** | **#GOs** | **GO IDs** | **Enzyme** | **NQPCT** | | |
|  |  |  |  |  |  |  |  |  |  |  |  | **Mf** | **Fw** | **Mw** |
| 94 | A8QEN2 | Bm1_51545 | 14992.m11090 | A8QEN2_BRUMA Melibiase family protein | S | 4 | Y | 0.71 | 3 | P:GO:0005975 F:GO:0003824 F:GO:0043169 | - | 0.87 | 0.00 | 0.07 |
| 95 | A8PVC0 | Bm1_35600 | 14968.m01509 | A8PVC0_BRUMA ML domain containing protein | S | 1 | Y | 0.58 | 4 | P:GO:0042632 C:GO:0005786 F:GO:0019899 C:GO:0005576 | - | 0.29 | 0.00 | 0.15 |
|  |  |  |  |  |  |  |  |  |  |  |  |  |  |  |
| **Proteins only found in Microfilariae** | | | | | | | | | | | | | | |
|  | **Uniprot ID** | **Pub_locus** | **TIGR locus** | **DESCRIPTION** | **TP** | **RC** | **SP** | **SecP** | **#GOs** | **GO IDs** | **Enzyme** | **NQPCT** | | |
|  |  |  |  |  |  |  |  |  |  |  |  | **Mf** | **Fw** | **Mw** |
| 96 | P29030 | Bm1_17035 | 14274.m00229 | CHIT_BRUMA Endochitinase precursor | S | 1 | Y | 0.53 | 11 | F:GO:0004568 P:GO:0006032 F:GO:0005509 P:GO:0009792 F:GO:0008061 C:GO:0005576 P:GO:0005975 P:GO:0006030 F:GO:0003824 F:GO:0043169 P:GO:0016998 | EC:3.2.1.14, | 13.04 | 0.00 | 0.00 |
| 97 | A8PJW0 | Bm1_28525 | 14931.m00318 | A8PJW0_BRUMA Serpin | S | 2 | Y | 0.49 | 1 | F:GO:0004867 |  | 9.42 | 0.00 | 0.00 |
| 98 | A8P664 | Bm1_17400 | 14293.m00075 | A8P664_BRUMA Trypsin inhibitor, putative | - | 1 | N | 0.69 | 3 | F:GO:0004867 F:GO:0004866 C:GO:0005576 | - | 2.61 | 0.00 | 0.00 |
| 99 | A8PVV9 | Bm1_35870 | 14971.m02814 | A8PVV9_BRUMA Copper type II ascorbate-dependent monooxygenase, C-terminal domain containing protein | S | 5 | N | 0.65 | 10 | P:GO:0006518 P:GO:0006697 F:GO:0035302 P:GO:0001700 P:GO:0048477 C:GO:0005783 F:GO:0005507 F:GO:0004500 P:GO:0006584 P:GO:0006570 | EC:1.14.17, | 2.03 | 0.00 | 0.00 |
| 100 | A8QFZ3 | Bm1_54345 | 15148.m00017 | A8QFZ3_BRUMA Zinc finger, C2H2 type family protein | - | 1 | N | 0.55 | 3 | F:GO:0003676 C:GO:0005622 F:GO:0008270 | - | 1.80 | 0.00 | 0.00 |
| 101 | A8PEN7 | Bm1_23665 | 14683.m00061 | A8PEN7_BRUMA Zinc finger, C2H2 type family protein | - | 3 | N | 0.80 | 3 | F:GO:0003676 C:GO:0005622 F:GO:0008270 | - | 1.59 | 0.00 | 0.00 |
| 102 | A8P605 | Bm1_17270 | 14284.m00379 | A8P605_BRUMA Fasciclin domain containing protein | - | 5 | N | 0.41 | 7 | P:GO:0001501 F:GO:0008201 P:GO:0007155 C:GO:0005578 F:GO:0005515 C:GO:0005576 C:GO:0005886 | - | 1.45 | 0.00 | 0.00 |
| 103 | A8PGG5 | Bm1_24780 | 14728.m00018 | A8PGG5_BRUMA Putative uncharacterized protein | - | 3 | N | 0.45 | 2 | F:GO:0016787 P:GO:0008152 |  | 1.45 | 0.00 | 0.00 |
| 104 | A8PLP4 | Bm1_29545 | 14946.m00531 | A8PLP4_BRUMA Zinc finger, C2H2 type family protein | - | 1 | N | 0.38 | 3 | F:GO:0003676 C:GO:0005622 F:GO:0008270 | - | 1.16 | 0.00 | 0.00 |
| 105 | O44932 | Bm1_14040 | 14046.m00191 | O44932_BRUMA Vespid allergen antigen homolog | S | 1 | Y | 0.59 | 1 | C:GO:0005576 |  | 1.16 | 0.00 | 0.00 |
| 106 | A8PJI2 | Bm1_27870 | 14920.m00403 | A8PJI2_BRUMA Putative uncharacterized protein | S | 2 | N | 0.47 | 0 |  | - | 1.01 | 0.00 | 0.00 |
| 107 | A8PHV1 | Bm1_25935 | 14777.m00108 | A8PHV1_BRUMA Serpin, putative | - | 3 | N | 0.65 | 1 | F:GO:0004867 |  | 0.43 | 0.00 | 0.00 |
| 108 | A8PQK5 | Bm1_31525 | 14956.m00486 | A8PQK5_BRUMA Probable zinc metalloproteinase, putative | - | 2 | N | 0.25 | 3 | P:GO:0006508 F:GO:0008533 F:GO:0008270 | - | 0.43 | 0.00 | 0.00 |
| 109 | A8PTF0 | Bm1_32290 | 14961.m04950 | A8PTF0_BRUMA Type I phosphodiesterase / nucleotide pyrophosphatase family protein | S | 2 | N | 0.62 | 6 | C:GO:0016020 P:GO:0044237 P:GO:0044238 P:GO:0048523 F:GO:0003676 F:GO:0004519 |  | 0.43 | 0.00 | 0.00 |
| 110 | A8Q7J6 | Bm1_45470 | 14979.m04611 | A8Q7J6_BRUMA Bromodomain containing protein | - | 1 | N | 0.25 | 11 | F:GO:0004674 P:GO:0007049 F:GO:0005515 F:GO:0004402 P:GO:0043283 F:GO:0003676 F:GO:0008270 P:GO:0009069 P:GO:0016310 C:GO:0000123 P:GO:0042967 | EC:2.7.11, EC:2.3.1.48, | 0.43 | 0.00 | 0.00 |
| 111 | A8QEU7 | Bm1_51805 | 14992.m11143 | A8QEU7_BRUMA Peptidase family M28 containing protein | S | 4 | Y | 0.68 | 2 | P:GO:0006508 F:GO:0008233 | - | 0.43 | 0.00 | 0.00 |
| 112 | A8QHJ5 | Bm1_57105 | 15480.m00009 | A8QHJ5_BRUMA Zinc finger, C2H2 type family protein | - | 1 | N | 0.91 | 3 | F:GO:0003676 C:GO:0005622 F:GO:0008270 | - | 0.43 | 0.00 | 0.00 |
| 113 | Q9NDV4 |  | - | Q9NDV4_BRUPA Mmc1 protein | S | 1 | Y | 0.76 | 0 |  | - | 0.43 | 0.00 | 0.00 |
| 114 | A8PDV2 | Bm1_22920 | 14650.m00141 | A8PDV2_BRUMA Putative uncharacterized protein | S | 1 | Y | 0.76 | 0 |  | - | 0.29 | 0.00 | 0.00 |
| 115 | A8QHP5 | Bm1_57355 | 15517.m00008 | A8QHP5_BRUMA Zinc finger, C2H2 type family protein | S | 3 | N | 0.66 | 3 | F:GO:0003676 C:GO:0005622 F:GO:0008270 | - | 0.29 | 0.00 | 0.00 |
| 116 | A8ND91 | Bm1_00290 | 12430.m00017 | A8ND91_BRUMA Zinc finger, C2H2 type family protein | - | 1 | N | 0.66 | 3 | F:GO:0003676 C:GO:0005622 F:GO:0008270 | - | 0.23 | 0.00 | 0.00 |
| 117 | A8NGT8 | Bm1_02240 | 12661.m00041 | A8NGT8_BRUMA Globin family protein | - | 1 | N | 0.71 | 4 | F:GO:0005506 F:GO:0020037 P:GO:0015671 F:GO:0019825 | - | 0.14 | 0.00 | 0.00 |
| 118 | A8NMN4 | Bm1_05700 | 13183.m00070 | A8NMN4_BRUMA Proteasome subunit alpha type 6, putative | - | 4 | N | 0.72 | 11 | F:GO:0003723 P:GO:0006511 P:GO:0009792 P:GO:0000003 F:GO:0004298 P:GO:0018996 P:GO:0002119 F:GO:0005515 P:GO:0040011 P:GO:0040015 C:GO:0005839 | EC:3.4.25, | 0.14 | 0.00 | 0.00 |
| 119 | A8NN46 | Bm1_06065 | 13224.m00016 | A8NN46_BRUMA Putative uncharacterized protein | - | 1 | N | 0.66 | 0 |  | - | 0.14 | 0.00 | 0.00 |
| 120 | A8NQJ7 | Bm1_07730 | 13330.m00017 | A8NQJ7_BRUMA Eukaryotic aspartyl protease family protein | - | 5 | Y | 0.50 | 4 | P:GO:0008219 F:GO:0005515 F:GO:0004194 P:GO:0006508 |  | 0.14 | 0.00 | 0.00 |
| 121 | A8NSY1 | Bm1_09110 | 13442.m00055 | A8NSY1_BRUMA Transmembrane GTPase fzo-1, putative | - | 3 | N | 0.35 | 7 | P:GO:0040010 P:GO:0009792 F:GO:0003924 F:GO:0005525 C:GO:0005741 P:GO:0008053 C:GO:0016021 |  | 0.14 | 0.00 | 0.00 |
| 122 | A8P773 | Bm1_18120 | 14349.m00107 | A8P773_BRUMA Putative uncharacterized protein | - | 3 | N | 0.54 | 8 | F:GO:0005488 F:GO:0005509 C:GO:0005737 P:GO:0007155 C:GO:0016020 P:GO:0007476 F:GO:0005515 C:GO:0005576 | - | 0.14 | 0.00 | 0.00 |
| 123 | A8PA11 | Bm1_20215 | 14496.m00119 | A8PA11_BRUMA Hsp90 protein | M | 1 | N | 0.47 | 9 | C:GO:0005615 P:GO:0040010 F:GO:0005164 C:GO:0005739 P:GO:0006457 F:GO:0051082 F:GO:0005524 P:GO:0006955 P:GO:0007165 |  | 0.14 | 0.00 | 0.00 |
| 124 | A8PHV4 | Bm1_25945 | 14777.m00110 | A8PHV4_BRUMA Serpin, putative | S | 1 | Y | 0.44 | 1 | F:GO:0004867 |  | 0.14 | 0.00 | 0.00 |
| 125 | A8PJM7 | Bm1_28090 | 14922.m00064 | A8PJM7_BRUMA Putative uncharacterized protein | S | 2 | Y | 0.87 | 0 |  | - | 0.14 | 0.00 | 0.00 |
| 126 | A8PJS8 | Bm1_28360 | 14929.m00397 | A8PJS8_BRUMA Phosphatidylinositol transfer protein | - | 1 | N | 0.34 | 9 | F:GO:0008526 C:GO:0016021 F:GO:0046872 C:GO:0044459 P:GO:0007608 F:GO:0008525 P:GO:0016059 C:GO:0005737 P:GO:0015914 |  | 0.14 | 0.00 | 0.00 |
| 127 | A8PXG7 | Bm1_37225 | 14972.m07138 | A8PXG7_BRUMA Calcium/calmodulin-dependent protein kinase type II alpha chain, putative | - | 2 | N | 0.54 | 18 | F:GO:0005516 C:GO:0030424 P:GO:0007528 C:GO:0030425 P:GO:0051489 C:GO:0005954 F:GO:0005524 P:GO:0007616 P:GO:0007268 P:GO:0040017 F:GO:0004713 C:GO:0045211 C:GO:0048786 P:GO:0040018 P:GO:0007619 F:GO:0004683 P:GO:0006468 P:GO:0009069 | EC:2.7.10, EC:2.7.11.17, | 0.14 | 0.00 | 0.00 |
| 128 | A8Q2E8 | Bm1_41135 | 14973.m02623 | A8Q2E8_BRUMA Abnormal embryonic partitioning of cytoplasm protein 3, isoform b, putative | - | 2 | N | 0.29 | 8 | P:GO:0051179 C:GO:0030054 P:GO:0000003 P:GO:0016043 P:GO:0048513 P:GO:0009653 C:GO:0044424 F:GO:0005515 |  | 0.14 | 0.00 | 0.00 |
| 129 | A8QCS7 | Bm1_49965 | 14990.m08119 | A8QCS7_BRUMA Putative uncharacterized protein | - | 2 | N | 0.46 | 1 | F:GO:0005515 |  | 0.14 | 0.00 | 0.00 |
| 130 | A8QF40 | Bm1_52270 | 14992.m11238 | A8QF40_BRUMA Putative uncharacterized protein | - | 2 | N | 0.64 | 3 | F:GO:0004182 P:GO:0006508 F:GO:0008270 | - | 0.14 | 0.00 | 0.00 |
|  |  |  |  |  |  |  |  |  |  |  |  |  |  |  |
| **Proteins only found in Female Worms** | | | | | | | | | | | | | | |
|  | **Uniprot ID** | **Pub_locus** | **TIGR locus** | **DESCRIPTION** | **TP** | **RC** | **SP** | **SecP** | **#GOs** | **GO IDs** | **Enzyme** | **NQPCT** | | |
|  |  |  |  |  |  |  |  |  |  |  |  | **Mf** | **Fw** | **Mw** |
| 131 | A8PIJ2 | Bm1_26615 | 14833.m00143 | A8PIJ2_BRUMA Insulin-degrading enzyme, putative | - | 1 | N | 0.33 | 4 | C:GO:0005615 F:GO:0004222 P:GO:0006508 F:GO:0008270 |  | 0.00 | 1.02 | 0.00 |
| 132 | A8NS73 | Bm1_08645 | 13404.m00015 | A8NS73_BRUMA Putative uncharacterized protein | - | 2 | N | 0.83 | 0 |  | - | 0.00 | 0.81 | 0.00 |
| 133 | A8PE26 | Bm1_23105 | 14654.m00080 | A8PE26_BRUMA A-macroglobulin complement component family protein | - | 1 | N | 0.55 | 4 | P:GO:0042742 F:GO:0004866 F:GO:0005515 C:GO:0005576 |  | 0.00 | 0.58 | 0.00 |
| 134 | A8PE24 | Bm1_23100 | 14654.m00079 | A8PE24_BRUMA Alpha-2-macroglobulin family N-terminal region containing protein | - | 2 | N | 0.74 | 5 | F:GO:0016740 P:GO:0008152 F:GO:0004866 F:GO:0005515 C:GO:0005576 | - | 0.00 | 0.41 | 0.00 |
| 135 | A8NND7 | Bm1_06185 | 13240.m00055 | A8NND7_BRUMA Papain family cysteine protease containing protein | - | 3 | N | 0.86 | 2 | F:GO:0004197 P:GO:0006508 | EC:3.4.22, | 0.00 | 0.34 | 0.00 |
| 136 | A8PN31 | Bm1_30225 | 14950.m01861 | A8PN31_BRUMA Dynein light chain 1, cytoplasmic, putative | - | 1 | N | 0.36 | 20 | F:GO:0003777 F:GO:0008092 P:GO:0040010 P:GO:0009792 C:GO:0005874 C:GO:0016459 F:GO:0042623 P:GO:0007476 P:GO:0040035 P:GO:0035046 P:GO:0007283 C:GO:0005868 P:GO:0010171 P:GO:0007018 P:GO:0002119 P:GO:0008039 P:GO:0040011 P:GO:0008407 P:GO:0002009 C:GO:000 |  | 0.00 | 0.27 | 0.00 |
| 137 | A8NLA3 | Bm1_04850 | 13066.m00247 | A8NLA3_BRUMA Hypothetical FAD-dependent oxidoreductase YEL047C, putative | S | 1 | Y | 0.54 | 6 | P:GO:0006118 F:GO:0000104 P:GO:0006119 P:GO:0018874 P:GO:0019643 C:GO:0045281 | - | 0.00 | 0.24 | 0.00 |
| 138 | A8NSE5 | Bm1_08800 | 13415.m00439 | A8NSE5_BRUMA Rab GDP dissociation inhibitor alpha, putative | - | 1 | N | 0.46 | 10 | P:GO:0016192 P:GO:0009792 P:GO:0008219 P:GO:0043087 C:GO:0008021 P:GO:0007269 F:GO:0005515 P:GO:0007165 F:GO:0005093 P:GO:0015031 |  | 0.00 | 0.24 | 0.00 |
| 139 | A8Q4D7 | Bm1_42865 | 14975.m04514 | A8Q4D7_BRUMA Antigen, putative | S | 1 | Y | 0.92 | 0 |  | - | 0.00 | 0.17 | 0.00 |
| 140 | A8QGR2 | Bm1_55695 | 15295.m00029 | A8QGR2_BRUMA Leucyl aminopeptidase, putative | - | 1 | N | 0.61 | 2 | F:GO:0004177 P:GO:0006508 | EC:3.4.11, | 0.00 | 0.17 | 0.00 |
| 141 | A8NJX0 | Bm1_04060 | 12923.m00023 | A8NJX0_BRUMA Malate dehydrogenase, cytoplasmic, putative | S | 1 | N | 0.36 | 13 | P:GO:0006107 C:GO:0005829 F:GO:0051287 F:GO:0030060 C:GO:0005625 P:GO:0006108 P:GO:0006100 F:GO:0004470 P:GO:0019674 P:GO:0006096 P:GO:0006090 P:GO:0019643 P:GO:0046487 | EC:1.1.1.37, | 0.00 | 0.14 | 0.00 |
| 142 | A8P3W1 | Bm1_15740 | 14208.m00924 | A8P3W1_BRUMA Phosphoglucomutase, putative | - | 2 | N | 0.43 | 11 | C:GO:0005737 F:GO:0046872 P:GO:0006874 F:GO:0004614 P:GO:0005982 P:GO:0005985 P:GO:0006012 P:GO:0006094 P:GO:0006096 P:GO:0006098 P:GO:0019872 | EC:5.4.2.2, | 0.00 | 0.14 | 0.00 |
| 143 | A8PM53 | Bm1_29765 | 14948.m00288 | A8PM53_BRUMA Excretory/secretory protein Juv-p120, putative | S | 1 | Y | 0.13 | 0 |  | - | 0.00 | 0.14 | 0.00 |
| 144 | A8Q165 | Bm1_36300 | 14972.m06949 | A8Q165_BRUMA Immunoglobulin I-set domain containing protein | - | 2 | N | 0.52 | 8 | F:GO:0005524 P:GO:0007399 F:GO:0005021 P:GO:0016358 C:GO:0016021 C:GO:0016020 P:GO:0001841 P:GO:0001756 | - | 0.00 | 0.14 | 0.00 |
| 145 | A8QDF5 | Bm1_52680 | 14992.m11319 | A8QDF5_BRUMA L-isoaspartate, putative | S | 1 | Y | 0.53 | 4 | P:GO:0006479 F:GO:0004719 F:GO:0005509 P:GO:0046500 | EC:2.1.1.77, | 0.00 | 0.14 | 0.00 |
| 146 | A8NF63 | Bm1_01300 | 12556.m00067 | A8NF63_BRUMA Alpha amylase, catalytic domain containing protein | - | 1 | N | 0.68 | 3 | F:GO:0003824 P:GO:0005975 F:GO:0043169 | - | 0.00 | 0.10 | 0.00 |
| 147 | A8PJV1 | Bm1_28480 | 14930.m00346 | A8PJV1_BRUMA Translation elongation factor aEF-2, putative | - | 1 | N | 0.39 | 12 | P:GO:0000022 C:GO:0005829 F:GO:0003746 P:GO:0009792 P:GO:0040007 P:GO:0040035 P:GO:0002119 F:GO:0003924 C:GO:0005811 F:GO:0005525 C:GO:0005840 P:GO:0006448 | EC:3.6.5.1, EC:3.6.5.2, EC:3.6.5.3, EC:3.6.5.4, | 0.00 | 0.10 | 0.00 |
| 148 | A8Q071 | Bm1_39250 | 14972.m07552 | A8Q071_BRUMA Protein disulphide isomerase, putative | S | 1 | Y | 0.34 | 18 | P:GO:0040010 F:GO:0003756 P:GO:0009792 F:GO:0004656 C:GO:0005793 P:GO:0045454 F:GO:0003810 C:GO:0009986 P:GO:0010171 C:GO:0005792 P:GO:0002119 F:GO:0005515 P:GO:0018401 P:GO:0040017 C:GO:0005576 C:GO:0005783 P:GO:0006525 P:GO:0006560 | EC:5.3.4.1, EC:1.14.11.2, EC:2.3.2.13, | 0.00 | 0.10 | 0.00 |
| 149 | A8Q1K1 | Bm1_36685 | 14972.m07028 | A8Q1K1_BRUMA Histone H4, putative | - | 1 | N | 0.41 | 11 | P:GO:0040035 P:GO:0010171 P:GO:0002119 C:GO:0000786 P:GO:0006952 F:GO:0003677 P:GO:0006334 P:GO:0040010 P:GO:0040011 P:GO:0009792 C:GO:0005634 |  | 0.00 | 0.10 | 0.00 |
| 150 | A8Q3Z6 | Bm1_42375 | 14975.m04412 | A8Q3Z6_BRUMA Heavy neurofilament protein, putative | S | 1 | Y | 0.36 | 0 |  | - | 0.00 | 0.10 | 0.00 |
| 151 | A8NDE0 | Bm1_00425 | 12442.m00022 | A8NDE0_BRUMA KMQK697, putative | - | 3 | N | 0.25 | 1 | C:GO:0009986 | - | 0.00 | 0.07 | 0.00 |
| 152 | A8NHQ1 | Bm1_02775 | 12737.m00157 | A8NHQ1_BRUMA 1,4-alpha-glucan branching enzyme, putative | - | 2 | N | 0.46 | 3 | F:GO:0016740 P:GO:0005975 F:GO:0043169 |  | 0.00 | 0.07 | 0.00 |
| 153 | A8NKX8 | Bm1_04575 | 13028.m00039 | A8NKX8_BRUMA Putative uncharacterized protein | M | 5 | N | 0.44 | 2 | C:GO:0005737 P:GO:0031424 | - | 0.00 | 0.07 | 0.00 |
| 154 | A8NUR6 | Bm1_10280 | 13579.m00041 | A8NUR6_BRUMA Transketolase, putative | - | 1 | N | 0.44 | 13 | P:GO:0040008 F:GO:0048029 C:GO:0005777 C:GO:0005625 F:GO:0004802 F:GO:0046872 P:GO:0009052 P:GO:0046390 C:GO:0005792 F:GO:0005515 C:GO:0005789 F:GO:0030976 P:GO:0015976 | EC:2.2.1.1, | 0.00 | 0.07 | 0.00 |
| 155 | A8PD34 | Bm1_22360 | 14619.m00057 | A8PD34_BRUMA DB module family protein | - | 1 | N | 0.62 | 0 |  | - | 0.00 | 0.07 | 0.00 |
| 156 | A8PHD7 | Bm1_25520 | 14765.m00814 | A8PHD7_BRUMA Putative uncharacterized protein |  |  | N |  | 4 | F:GO:0005524 F:GO:0008134 F:GO:0016887 C:GO:0005667 |  | 0.00 | 0.07 | 0.00 |
| 157 | A8PIM8 | Bm1_26725 | 14851.m00011 | A8PIM8_BRUMA Proteasome subunit alpha type 1, putative | - | 2 | N | 0.35 | 4 | F:GO:0005515 F:GO:0004298 C:GO:0005839 P:GO:0006511 |  | 0.00 | 0.07 | 0.00 |
| 158 | A8PJH0 | Bm1_27810 | 14919.m00204 | A8PJH0_BRUMA Nucleosome assembly protein 1, putative | - | 1 | N | 0.33 | 10 | P:GO:0040035 P:GO:0010171 P:GO:0002119 P:GO:0006334 F:GO:0005515 P:GO:0040010 P:GO:0040011 P:GO:0009792 C:GO:0005634 P:GO:0002009 |  | 0.00 | 0.07 | 0.00 |
| 159 | A8Q2A0 | Bm1_40960 | 14973.m02730 | A8Q2A0_BRUMA Chymotrypsin/elastase isoinhibitors 2 to 5, putative | S | 1 | Y | 0.91 | 1 | F:GO:0008233 |  | 0.00 | 0.07 | 0.00 |
| 160 | A8QE01 | Bm1_50525 | 14992.m10877 | A8QE01_BRUMA Ube1-prov protein, putative | - | 1 | N | 0.51 | 5 | F:GO:0008641 F:GO:0005515 P:GO:0000003 P:GO:0009792 P:GO:0006512 |  | 0.00 | 0.07 | 0.00 |
| 161 | A8QE32 | Bm1_50655 | 14992.m10905 | A8QE32_BRUMA Galactoside-binding lectin family protein | - | 4 | N | 0.56 | 1 | F:GO:0005529 | - | 0.00 | 0.07 | 0.00 |
| 162 | A1BQ63 | Bm1_31480 | 14955.m00256 | A1BQ63_BRUMA Translationally controlled tumor protein-like protein | - | 1 | N | 0.71 | 7 | C:GO:0005737 P:GO:0000003 P:GO:0009792 P:GO:0040010 F:GO:0005509 F:GO:0005515 P:GO:0040011 |  | 0.00 | 0.03 | 0.00 |
| 163 | A8NPI1 | Bm1_07000 | 13282.m00270 | A8NPI1_BRUMA Serine protease Z688.6, putative | - | 3 | N | 0.40 | 3 | P:GO:0009792 F:GO:0008236 P:GO:0006508 |  | 0.00 | 0.03 | 0.00 |
| 164 | A8NQH2 | Bm1_07640 | 13322.m00195 | A8NQH2_BRUMA Heat shock 70 kDa protein C, putative | - | 1 | N | 0.39 | 14 | P:GO:0006983 P:GO:0009306 F:GO:0005509 P:GO:0040007 F:GO:0043022 C:GO:0042470 F:GO:0005524 P:GO:0010171 P:GO:0002119 C:GO:0005788 F:GO:0016887 F:GO:0005515 P:GO:0040011 P:GO:0030968 |  | 0.00 | 0.03 | 0.00 |
| 165 | A8NRT7 | Bm1_08465 | 13390.m00016 | A8NRT7_BRUMA Putative uncharacterized protein | - | 2 | N | 0.65 | 0 |  | - | 0.00 | 0.03 | 0.00 |
| 166 | A8NWB4 | Bm1_11280 | 13695.m00212 | A8NWB4_BRUMA GDP-L-fucose synthetase, putative | - | 3 | N | 0.35 | 8 | F:GO:0050577 P:GO:0042350 F:GO:0050662 F:GO:0019787 P:GO:0043687 P:GO:0051246 P:GO:0006000 P:GO:0006013 | EC:1.1.1.271, | 0.00 | 0.03 | 0.00 |
| 167 | A8NWE7 | Bm1_11390 | 13714.m00023 | A8NWE7_BRUMA Protein C37C3.6a , putative | - | 1 | N | 0.61 | 10 | P:GO:0040035 P:GO:0002119 F:GO:0005488 P:GO:0040002 P:GO:0040010 P:GO:0040011 P:GO:0009792 P:GO:0008340 P:GO:0002009 F:GO:0004867 |  | 0.00 | 0.03 | 0.00 |
| 168 | A8P026 | Bm1_13165 | 13960.m00025 | A8P026_BRUMA Putative uncharacterized protein | - | 2 | N | 0.47 | 1 | P:GO:0040011 | - | 0.00 | 0.03 | 0.00 |
| 169 | A8P1B6 | Bm1_14055 | 14046.m00194 | A8P1B6_BRUMA ShTK domain containing protein | - | 1 | N | 0.86 | 1 | F:GO:0005515 | - | 0.00 | 0.03 | 0.00 |
| 170 | A8P4C6 | Bm1_16060 | 14231.m00144 | A8P4C6_BRUMA Carbohydrate phosphorylase, putative | M | 4 | N | 0.40 | 12 | F:GO:0008184 C:GO:0016529 F:GO:0008144 P:GO:0000910 P:GO:0005980 P:GO:0009792 P:GO:0006874 F:GO:0042803 F:GO:0016208 F:GO:0030170 P:GO:0051591 F:GO:0005529 |  | 0.00 | 0.03 | 0.00 |
| 171 | A8P7M6 | Bm1_18460 | 14364.m00213 | A8P7M6_BRUMA Phosphoglucomutase/phosphomannomutase, C-terminal domain containing protein | - | 2 | N | 0.74 | 6 | P:GO:0005975 F:GO:0016868 P:GO:0040010 P:GO:0009792 F:GO:0005515 P:GO:0002119 | EC:5.4.2, | 0.00 | 0.03 | 0.00 |
| 172 | A8P9L4 | Bm1_19890 | 14469.m00105 | A8P9L4_BRUMA Phosphoribosyl transferase domain containing protein | - | 1 | N | 0.88 | 19 | P:GO:0019835 P:GO:0006168 P:GO:0045964 P:GO:0051289 P:GO:0001975 P:GO:0021954 P:GO:0006178 P:GO:0007625 F:GO:0004422 P:GO:0021895 P:GO:0021756 P:GO:0048813 P:GO:0046651 C:GO:0005737 P:GO:0046100 F:GO:0000287 P:GO:0006164 P:GO:0006166 F:GO:0042803 | EC:2.4.2.8, | 0.00 | 0.03 | 0.00 |
| 173 | A8P9X6 | Bm1_20115 | 14489.m00059 | A8P9X6_BRUMA Putative uncharacterized protein | - | 2 | N | 0.38 | 0 |  | - | 0.00 | 0.03 | 0.00 |
| 174 | A8PAN8 | Bm1_20640 | 14529.m00201 | A8PAN8_BRUMA Galactoside-binding lectin family protein | - | 4 | N | 0.49 | 2 | F:GO:0005515 F:GO:0016936 |  | 0.00 | 0.03 | 0.00 |
| 175 | A8PAY9 | Bm1_20880 | 14541.m00145 | A8PAY9_BRUMA Phosphoglycerate mutase family protein | - | 3 | N | 0.43 | 0 |  | - | 0.00 | 0.03 | 0.00 |
| 176 | A8PDX6 | Bm1_22990 | 14652.m00402 | A8PDX6_BRUMA Actin-depolymerizing factor 1, putative | - | 2 | N | 0.88 | 9 | P:GO:0030042 C:GO:0030016 P:GO:0030240 F:GO:0051015 P:GO:0040035 P:GO:0002119 P:GO:0009790 P:GO:0040011 C:GO:0005884 |  | 0.00 | 0.03 | 0.00 |
| 177 | A8PGH9 | Bm1_24805 | 14731.m00984 | A8PGH9_BRUMA Proteasome subunit beta type 3, putative | - | 3 | N | 0.47 | 5 | C:GO:0005839 F:GO:0005515 P:GO:0006510 F:GO:0004298 P:GO:0006511 |  | 0.00 | 0.03 | 0.00 |
| 178 | A8PJI5 | Bm1_27885 | 14920.m00406 | A8PJI5_BRUMA Peptidase family M1 containing protein | - | 2 | N |  | 10 | C:GO:0005737 P:GO:0009792 F:GO:0004179 P:GO:0006508 F:GO:0005524 P:GO:0006457 F:GO:0051082 F:GO:0008270 P:GO:0006749 C:GO:0016020 | EC:3.4.11, | 0.00 | 0.03 | 0.00 |
| 179 | A8PN33 | Bm1_30230 | 14950.m01862 | A8PN33_BRUMA Hypothetical 19.4 kDa protein ZC395.10 in chromosome III, putative | - | 3 | N | 0.23 | 2 | P:GO:0040010 P:GO:0009792 |  | 0.00 | 0.03 | 0.00 |
| 180 | A8PNF4 | Bm1_30360 | 14952.m01370 | A8PNF4_BRUMA Cathepsin Z-like cysteine proteinase, putative | S | 2 | Y | 0.70 | 8 | P:GO:0010171 P:GO:0002119 C:GO:0031012 P:GO:0018996 P:GO:0009792 F:GO:0004197 P:GO:0040007 P:GO:0006508 | EC:3.4.22, | 0.00 | 0.03 | 0.00 |
| 181 | A8PNV8 | Bm1_30540 | 14954.m01572 | A8PNV8_BRUMA Glycosyl hydrolases family 38 protein | - | 3 | N | 0.47 | 3 | F:GO:0004559 F:GO:0005515 P:GO:0006013 | EC:3.2.1.24, | 0.00 | 0.03 | 0.00 |
| 182 | A8PPA3 | Bm1_30715 | 14954.m01607 | A8PPA3_BRUMA Putative uncharacterized protein | S | 3 | Y | 0.94 | 0 |  | - | 0.00 | 0.03 | 0.00 |
| 183 | A8PQR8 | Bm1_31775 | 14956.m00536 | A8PQR8_BRUMA ARMET-like protein, putative | S | 1 | Y | 0.88 | 2 | C:GO:0005615 F:GO:0005488 |  | 0.00 | 0.03 | 0.00 |
| 184 | A8PXM0 | Bm1_37310 | 14972.m07156 | A8PXM0_BRUMA FKBP-type peptidyl-prolyl cis-trans isomerase-13, BmFKBP-13 | S | 1 | Y | 0.76 | 4 | C:GO:0005789 C:GO:0005615 P:GO:0006457 F:GO:0003755 | EC:5.2.1.8, | 0.00 | 0.03 | 0.00 |
| 185 | A8PXR9 | Bm1_37390 | 14972.m07172 | A8PXR9_BRUMA 20S proteasome alpha5 subunit, putative | - | 2 | N | 0.38 | 11 | P:GO:0002119 C:GO:0005839 F:GO:0005515 P:GO:0006511 F:GO:0004298 P:GO:0000003 P:GO:0040011 P:GO:0009792 P:GO:0040007 F:GO:0004866 C:GO:0005634 | EC:3.4.25, | 0.00 | 0.03 | 0.00 |
| 186 | A8Q131 | Bm1_40615 | 14972.m07910 | A8Q131_BRUMA Putative uncharacterized protein | - | 5 | N | 0.12 | 0 |  | - | 0.00 | 0.03 | 0.00 |
| 187 | A8Q7C7 | Bm1_45210 | 14979.m04551 | A8Q7C7_BRUMA Ubiquitin-like protein SMT3, putative | - | 2 | N | 0.67 | 23 | P:GO:0040010 P:GO:0006281 P:GO:0009792 P:GO:0016925 C:GO:0030425 P:GO:0043433 P:GO:0040002 P:GO:0032880 C:GO:0045202 C:GO:0016607 C:GO:0005643 P:GO:0040027 P:GO:0040035 F:GO:0004842 P:GO:0010171 C:GO:0016605 F:GO:0008134 F:GO:0019789 P:GO:0016481 P:GO:000 | EC:6.3.2.19, | 0.00 | 0.03 | 0.00 |
| 188 | A8Q7G8 | Bm1_45370 | 14979.m04586 | A8Q7G8_BRUMA Cullin family protein | - | 4 | N | 0.33 | 12 | P:GO:0008054 P:GO:0009792 P:GO:0042078 P:GO:0051232 C:GO:0005737 P:GO:0008105 P:GO:0008595 C:GO:0005634 P:GO:0051759 P:GO:0007138 C:GO:0031461 F:GO:0031625 |  | 0.00 | 0.03 | 0.00 |
| 189 | A8QE02 | Bm1_50530 | 14992.m10878 | A8QE02_BRUMA Aminopeptidase W07G4.4 in chromosome V, putative | - | 4 | N | 0.49 | 4 | F:GO:0016806 C:GO:0005764 F:GO:0004177 P:GO:0006508 | EC:3.4.14, EC:3.4.11, | 0.00 | 0.03 | 0.00 |
| 190 | A8QEY1 | Bm1_51975 | 14992.m11177 | A8QEY1_BRUMA Probable complex I intermediate-associated protein 30, mitochondrialprecursor., putative | S | 3 | Y | 0.39 | 2 | F:GO:0003676 F:GO:0000166 | - | 0.00 | 0.03 | 0.00 |
| 191 | A8QFH2 | Bm1_53445 | 15048.m00015 | A8QFH2_BRUMA Putative uncharacterized protein | - | 3 | N | 0.21 | 0 |  | - | 0.00 | 0.03 | 0.00 |
| 192 | A8QGG4 | Bm1_55200 | 15233.m00030 | A8QGG4_BRUMA Proteasome A-type and B-type family protein | - | 1 | N | 0.45 | 11 | P:GO:0006511 P:GO:0009401 P:GO:0009792 P:GO:0040007 F:GO:0004298 P:GO:0000003 P:GO:0018996 P:GO:0002119 P:GO:0040011 F:GO:0005351 C:GO:0005839 | EC:3.4.25, | 0.00 | 0.03 | 0.00 |
| 193 | A8QGL9 | Bm1_55480 | 15264.m00007 | A8QGL9_BRUMA Transitional endoplasmic reticulum ATPase TER94, putative | - | 2 | N | 0.52 | 22 | P:GO:0008103 P:GO:0009792 F:GO:0005515 F:GO:0017111 P:GO:0007029 P:GO:0040010 P:GO:0040018 P:GO:0042052 P:GO:0040011 P:GO:0007030 P:GO:0040002 P:GO:0007317 P:GO:0016320 C:GO:0045169 P:GO:0008104 C:GO:0000502 P:GO:0002119 P:GO:0006508 F:GO:0008233 C:GO:000 | EC:3.6.1.15, | 0.00 | 0.03 | 0.00 |
| 194 | A8QHE7 | Bm1_56870 | 15451.m00017 | A8QHE7_BRUMA FKBP-type peptidyl-prolyl cis-trans isomerase-59, BmFKBP59 | - | 1 | N | 0.22 | 20 | F:GO:0030674 F:GO:0031072 P:GO:0006825 P:GO:0009792 P:GO:0006457 F:GO:0035259 P:GO:0007566 P:GO:0031503 P:GO:0006463 F:GO:0005528 F:GO:0005524 P:GO:0046661 F:GO:0051219 F:GO:0016853 P:GO:0002009 C:GO:0005737 C:GO:0005634 F:GO:0005525 F:GO:0005488 P:GO:000 |  | 0.00 | 0.03 | 0.00 |
| 195 | Q6H323 | Bm1_28935 | 14937.m00487 | Q6H323_BRUMA Transglutaminase | - | 1 | N | 0.21 | 15 | F:GO:0047961 F:GO:0003756 P:GO:0018991 P:GO:0008360 P:GO:0040002 P:GO:0045454 P:GO:0045138 F:GO:0003810 P:GO:0010171 C:GO:0005811 P:GO:0040018 C:GO:0005783 P:GO:0040019 F:GO:0016853 P:GO:0042967 | EC:2.3.1.13, EC:5.3.4.1, EC:2.3.2.13, | 0.00 | 0.03 | 0.00 |
| 196 | Q75PZ6 | Bm1_23315 | 14665.m00020 | Q75PZ6_BRUMA Cahepsin L-like non-peptiedase homolog | S | 2 | Y | 0.87 | 3 | F:GO:0004197 P:GO:0006508 F:GO:0008234 | EC:3.4.22, | 0.00 | 0.03 | 0.00 |
| 197 | Q9GU91 | Bm1_33050 | 14961.m05101 | Q9GU91_BRUMA Embryonic fatty acid-binding protein Bm-FAB-1 precursor | S | 1 | Y | 0.67 | 5 | P:GO:0006810 F:GO:0005515 F:GO:0008289 F:GO:0005215 F:GO:0005488 |  | 0.00 | 0.03 | 0.00 |
|  |  |  |  |  |  |  |  |  |  |  |  |  |  |  |
| **Proteins only found in Male worms** | | | | | | | | | | | | | | |
|  | **Uniprot ID** | **Pub_locus** | **TIGR locus** | **DESCRIPTION** | **TP** | **RC** | **SP** | **SecP** | **#GOs** | **GO IDs** | **Enzyme** | **NQPCT** | | |
|  |  |  |  |  |  |  |  |  |  |  |  | **Mf** | **Fw** | **Mw** |
| 198 | A8P5A0 | Bm1_16810 | 14258.m00140 | A8P5A0_BRUMA Actin, putative | - | 1 | N | 0.50 | 11 | C:GO:0005737 F:GO:0005200 P:GO:0040035 C:GO:0005856 P:GO:0002119 P:GO:0030036 F:GO:0005515 P:GO:0000910 F:GO:0005524 P:GO:0009792 P:GO:0040007 |  | 0.00 | 0.00 | 1.24 |
| 199 | A8PC53 | Bm1_21705 | 14594.m00163 | A8PC53_BRUMA Actin 1, putative | - | 1 | N | 0.50 | 14 | P:GO:0007291 P:GO:0000910 P:GO:0009792 P:GO:0040007 P:GO:0040035 P:GO:0030036 P:GO:0006911 F:GO:0005524 P:GO:0002119 F:GO:0005515 C:GO:0031011 C:GO:0005884 F:GO:0005200 C:GO:0005811 |  | 0.00 | 0.00 | 1.02 |
| 200 | A8NW97 | Bm1_11195 | 13687.m00015 | A8NW97_BRUMA P40, putative | - | 1 | N | 0.36 | 0 |  | - | 0.00 | 0.00 | 0.88 |
| 201 | O76497 | Bm1_23480 | 14674.m00132 | O76497_BRUMA 24 kDa secreted protein | S | 2 | Y | 0.92 | 0 |  | - | 0.00 | 0.00 | 0.81 |
| 202 | A8Q7K7 | Bm1_45510 | 14979.m04621 | A8Q7K7_BRUMA MFP3, putative | - | 3 | N | 0.56 | - | - | - | 0.00 | 0.00 | 0.51 |
| 203 | A8PV46 | Bm1_35295 | 14968.m01452 | A8PV46_BRUMA Immunoglobulin I-set domain containing protein | - | 2 | N | 0.54 | 2 | F:GO:0016301 F:GO:0005515 |  | 0.00 | 0.00 | 0.44 |
| 204 | A8P5F6 | Bm1_16920 | 14269.m00019 | A8P5F6_BRUMA Major sperm protein 3, putative | - | 1 | N | 0.34 | 4 | P:GO:0040017 P:GO:0040010 F:GO:0005515 F:GO:0005198 |  | 0.00 | 0.00 | 0.37 |
| 205 | A8NU76 | Bm1_09940 | 13528.m00031 | A8NU76_BRUMA P40, putative | - | 1 | N | 0.27 | 0 |  | - | 0.00 | 0.00 | 0.29 |
| 206 | A8QF97 | Bm1_52555 | 14992.m11294 | A8QF97_BRUMA G2F domain containing protein | S | 1 | Y | 0.58 | 5 | C:GO:0016020 F:GO:0005509 P:GO:0007160 P:GO:0008218 P:GO:0018298 |  | 0.00 | 0.00 | 0.29 |
| 207 | A8QGS4 | Bm1_55755 | 15304.m00111 | A8QGS4_BRUMA Major sperm protein, putative | - | 2 | N | 0.73 | 3 | C:GO:0005856 F:GO:0005198 F:GO:0005515 |  | 0.00 | 0.00 | 0.29 |
| 208 | A8PHN9 | Bm1_25755 | 14773.m00913 | A8PHN9_BRUMA Putative uncharacterized protein | - | 2 | N | 0.60 | 2 | F:GO:0005515 P:GO:0008340 |  | 0.00 | 0.00 | 0.22 |
| 209 | A8Q5X7 | Bm1_43615 | 14977.m04971 | A8Q5X7_BRUMA Cytoplasmic intermediate filament protein, putative | - | 1 | N | 0.80 | 12 | C:GO:0005737 P:GO:0010171 P:GO:0002119 P:GO:0018996 F:GO:0005515 P:GO:0040002 C:GO:0005882 P:GO:0040010 P:GO:0040011 P:GO:0009792 P:GO:0008340 F:GO:0005198 |  | 0.00 | 0.00 | 0.22 |
| 210 | A8NWI1 | Bm1_11450 | 13718.m00044 | A8NWI1_BRUMA Chaperonin homolog HSP60, mitochondrial, putative | - | 2 | N | 0.33 | 10 | P:GO:0042026 P:GO:0009792 P:GO:0000003 P:GO:0006626 P:GO:0009408 F:GO:0005524 C:GO:0005759 F:GO:0051082 P:GO:0006458 C:GO:0005811 |  | 0.00 | 0.00 | 0.15 |
| 211 | A8P0Z7 | Bm1_13790 | 14037.m00196 | A8P0Z7_BRUMA Adenylate kinase isoenzyme 1, putative | S | 5 | N | 0.70 | 8 | F:GO:0004017 P:GO:0007050 P:GO:0046034 C:GO:0005886 F:GO:0005515 F:GO:0005524 C:GO:0005739 P:GO:0006144 | EC:2.7.4.3, | 0.00 | 0.00 | 0.15 |
| 212 | A8Q5Q1 | Bm1_43360 | 14977.m04916 | A8Q5Q1_BRUMA Putative uncharacterized protein | S | 1 | Y | 0.61 | 0 |  | - | 0.00 | 0.00 | 0.15 |
| 213 | A8Q5X1 | Bm1_43595 | 14977.m04966 | A8Q5X1_BRUMA Putative uncharacterized protein | - | 5 | N | 0.22 | 1 | F:GO:0005488 | - | 0.00 | 0.00 | 0.15 |
| 214 | Q17181 | Bm1_45215 | 14979.m04552 | Q17181_BRUMA Intermediate filament protein | - | 1 | N | 0.48 | 11 | C:GO:0005737 P:GO:0010171 P:GO:0002119 P:GO:0018991 P:GO:0018996 F:GO:0005515 C:GO:0005882 P:GO:0040010 P:GO:0040011 P:GO:0009792 F:GO:0005198 |  | 0.00 | 0.00 | 0.15 |
| 215 | A8NMB0 | Bm1_05440 | 13154.m00138 | A8NMB0_BRUMA Thioredoxin family protein | - | 2 | N | 0.49 | 1 | P:GO:0045454 | - | 0.00 | 0.00 | 0.07 |
| 216 | A8NQ59 | Bm1_07405 | 13314.m00593 | A8NQ59_BRUMA WD40-like Beta Propeller Repeat family protein | S | 2 | N | 0.70 | 1 | F:GO:0004177 | - | 0.00 | 0.00 | 0.07 |
| 217 | A8NWA3 | Bm1_11225 | 13693.m00017 | A8NWA3_BRUMA Heparan sulfate, putative | - | 2 | N | 0.68 | 4 | P:GO:0009987 P:GO:0007517 C:GO:0031012 C:GO:0044421 |  | 0.00 | 0.00 | 0.07 |
| 218 | A8P1B1 | Bm1_14035 | 14046.m00190 | A8P1B1_BRUMA SCP-like extracellular protein | S | 2 | Y | 0.71 | 1 | C:GO:0005576 |  | 0.00 | 0.00 | 0.07 |
| 219 | A8PGZ3 | Bm1_25195 | 14749.m00214 | A8PGZ3_BRUMA Phosphoenolpyruvate carboxykinase [GTP] , putative | S | 5 | N | 0.54 | 4 | P:GO:0006094 F:GO:0005525 F:GO:0004613 P:GO:0006099 | EC:4.1.1.32, | 0.00 | 0.00 | 0.07 |
| 220 | A8PHJ7 | Bm1_25670 | 14771.m00162 | A8PHJ7_BRUMA Putative uncharacterized protein | - | 3 | N | 0.18 | 5 | P:GO:0007519 P:GO:0048468 P:GO:0007391 P:GO:0016043 C:GO:0044444 |  | 0.00 | 0.00 | 0.07 |
| 221 | A8PJG3 | Bm1_27775 | 14919.m00197 | A8PJG3_BRUMA Cyanate hydratase family protein | - | 4 | N | 0.52 | 4 | F:GO:0008824 P:GO:0009439 F:GO:0016836 F:GO:0003677 |  | 0.00 | 0.00 | 0.07 |
| 222 | A8PY76 | Bm1_37920 | 14972.m07279 | A8PY76_BRUMA Egg laying defective protein 4, isoform c, putative | - | 2 | N | 0.39 | 15 | P:GO:0001764 P:GO:0016358 P:GO:0030900 F:GO:0004692 F:GO:0005524 F:GO:0008603 F:GO:0005515 F:GO:0004713 P:GO:0007165 F:GO:0030553 C:GO:0005952 C:GO:0005794 P:GO:0006468 P:GO:0009069 P:GO:0045859 | EC:2.7.11.12, EC:2.7.10, | 0.00 | 0.00 | 0.07 |
| 223 | A8Q1S9 | Bm1_36910 | 14972.m07073 | A8Q1S9_BRUMA Putative uncharacterized protein | - | 1 | N | 0.71 | 0 |  | - | 0.00 | 0.00 | 0.07 |
| 224 | A8Q2J5 | Bm1_41320 | 14973.m02656 | A8Q2J5_BRUMA UDP-glucose pyrophosphorylase, putative | - | 1 | N | 0.41 | 11 | C:GO:0005737 F:GO:0042802 P:GO:0002119 P:GO:0040010 F:GO:0003983 P:GO:0009792 P:GO:0006355 P:GO:0005982 P:GO:0005985 P:GO:0006012 P:GO:0009117 | EC:2.7.7.9, | 0.00 | 0.00 | 0.07 |
| 225 | A8QCC8 | Bm1_49465 | 14990.m08019 | A8QCC8_BRUMA Proteasome subunit alpha type 7-1, putative | - | 2 | N | 0.28 | 11 | P:GO:0040010 P:GO:0006511 F:GO:0004866 P:GO:0009792 P:GO:0000003 F:GO:0004298 F:GO:0042802 P:GO:0002119 P:GO:0040011 C:GO:0005839 C:GO:0005634 | EC:3.4.25, | 0.00 | 0.00 | 0.07 |
| 226 | A8QFE1 | Bm1_53295 | 15018.m00015 | A8QFE1_BRUMA Putative uncharacterized protein | S | 1 | Y | 0.81 | 0 |  | - | 0.00 | 0.00 | 0.07 |
| 227 | A8QFL6 | Bm1_53680 | 15077.m00106 | A8QFL6_BRUMA Putative uncharacterized protein | - | 3 | Y | 0.49 | 0 |  | - | 0.00 | 0.00 | 0.07 |
| 228 | A8QH89 | Bm1_56580 | 15413.m00008 | A8QH89_BRUMA Chaperonine protein HSP60, putative | M | 3 | N | 0.53 | 33 | C:GO:0045121 P:GO:0000003 P:GO:0032496 F:GO:0051087 P:GO:0009792 P:GO:0042542 P:GO:0006458 F:GO:0043559 C:GO:0005759 P:GO:0033198 C:GO:0030141 C:GO:0005743 P:GO:0043627 C:GO:0005794 P:GO:0045768 C:GO:0005829 F:GO:0046982 P:GO:0001666 F:GO:0051082 P:GO:004 |  | 0.00 | 0.00 | 0.07 |
