# Supplementary material for: Stage- and Gender-Specific Proteomic Analysis of Brugia malayi Excretory-Secretory Products
Source: PLoS Negl Trop Dis. 2008 Oct 29;2(10):e326. doi: 10.1371/journal.pntd.0000326 (PMC2569413; doi:10.1371/journal.pntd.0000326)
Supplement: Table S2 — List of previously secreted proteins identified by other methods in B. malayi and other parasitic nematode species. (0.11 MB DOC) [file pntd.0000326.s004.doc]

| **Previously identified in *Brugia* Found in this work** |  | **Reference** |
| --- | --- | --- |
| Glutathione peroxidase (Bm-GPX-1) |  | [1-3] |
| Cystatin (Bm-CPI-2) |  | [4] |
| Serpin (Bm-SPN-1) |  | [5] |
| Serpin (Bm-SPN-2) |  | [6] |
| Macrophage Migration Inhibitory Factor (Bm-MIF-1) |  | [7] |
| Macrophage Migration Inhibitory Factor (Bm-MIF-2) |  | [8] |
| Superoxide dismutase CuZn (Bm-SOD) |  | [9] |
| Vespid venom allergen (Bm-VAL-1) |  | [10] |
| Glutathione S-transferase (Bm-GST) |  | [11] |
| 17/200 kDa ladder antigen |  | [2] |
|  |  |  |
| **Previously reported but not found** |  |  |
| Transforming Growth Factor (Bm-TGH-2) |  | [12] |
| Acetylcholinesterase |  | [13] |
|  |  |  |
| **Found in other parasitic nematodes** | **Species** |  |
| 22 kDa secreted protein / Alt-1 | Dirofilaria immitis/B. pahangi | [14] |
| Leucine Aminopeptidase | Acanthocheilonema viteae | [15] |
| Ov16 | Onchocerca volvulus | [16] |
| Ov20/Bm20 | Onchocerca volvulus/B. malayi | [17] |
| Chitinase | O. volvulus/A. viteae | [18] |
| Excretory/secretory antigen (Juv-p120) | Litomosoides sigmodontis | [19] |
|  |  |  |
| **Reported to be not present in *Brugia* ESP but identified in this work** |  |  |
| Thioredoxin |  | [20] |
|  |  |  |

**Table S2**

**List of previously secreted proteins identified by other methods in *B. malayi* and other parasitic nematode species**

**References Table S1**

1. Cookson E, Blaxter ML, Selkirk ME (1992) Identification of the major soluble cuticular glycoprotein of lymphatic filarial nematode parasites (gp29) as a secretory homolog of glutathione peroxidase. Proceedings of the National Academy of Sciences of the United States of America 89: 5837-5841.

2. Maizels RM, Gregory WF, Kwan-Lim GE, Selkirk ME (1988) Filarial surface antigens: the major 29 kilodalton glycoprotein and a novel 17-200 kilodalton complex from adult *Brugia malayi* parasites. Molecular and Biochemical Parasitology 32: 213-228.

3. Tang L, Smith VP, Gounaris K, Selkirk ME (1996) *Brugia pahangi*: The cuticular glutathione peroxidase (gp29) protects heterologous membranes from lipid peroxidation. Experimental Parasitology 82: 329-332.

4. Manoury B, Gregory WF, Maizels RM, Watts C (2001) Bm-CPI-2, a cystatin homolog secreted by the filarial parasite *Brugia malayi*, inhibits class II MHC-restricted antigen processing. Current Biology 11: 447-451.

5. Yenbutr P, Scott AL (1995) Molecular cloning of a serine proteinase inhibitor from Brugia malayi. Infection and Immunity 63: 1745-1753.

6. Zang X, Atmadja AK, Gray P, Allen JE, Gray CA, et al. (2000) The serpin secreted by *Brugia malayi* microfilariae, Bm-SPN-2, elicits strong, but short-lived, immune responses in mice and humans. Journal of Immunology 165: 5161-5169.

7. Pastrana DV, Raghavan N, Fitzgerald P, Eisinger SW, Metz C, et al. (1998) Filarial nematode parasites secrete a homologue of the human cytokine macrophage migration inhibitory factor. Infection and Immunity 66: 5955-5963.

8. Zang X, Taylor P, Wang JM, Meyer DJ, Scott AL, et al. (2002) Homologues of human macrophage migration inhibitory factor from a parasitic nematode: Gene cloning, protein activity, and crystal structure. Journal of Biological Chemistry 277: 44261-44267.

9. Tang L, Ou X, Henkle-Duhrsen K, Selkirk ME (1994) Extracellular and cytoplasmic CuZn superoxide dismutases from *Brugia* lymphatic filarial nematode parasites. Infection and Immunity 62: 961-967.

10. Murray J, Gregory WF, Gomez-Escobar N, Atmadja AK, Maizels RM (2001) Expression and immune recognition of *Brugia malayi* VAL-1, a homologue of vespid venom allergens and *Ancylostoma* secreted proteins. Molecular and Biochemical Parasitology 118: 89-96.

11. Rao UR, Salinas G, Mehta K, Klei TR (2000) Identification and localization of glutathione S-transferase as a potential target enzyme in *Brugia* species. Parasitology Research 86: 908-915.

12. Gomez-Escobar N, Gregory WF, Maizels RM (2000) Identification of tgh-2, a filarial nematode homolog of *Caenorhabditis elegans* daf-7 and human transforming growth factor β, expressed in microfilarial and adult stages of *Brugia malayi*. Infection and Immunity 68: 6402-6410.

13. Rathaur S, Robertson BD, Selkirk ME, Maizels RM (1987) Secretory acetylcholinesterases from *Brugia malayi* adult and microfilarial parasites. Molecular and Biochemical Parasitology 26: 257-265.

14. Frank GR, Tripp CA, Grieve RB (1996) Molecular cloning of a developmentally regulated protein isolated from excretory-secretory products of larval *Dirofilaria immitis*. Molecular and Biochemical Parasitology 75: 231-240.

15. Harnett W, Houston KM, Tate R, Garate T, Apfel H, et al. (1999) Molecular cloning and demonstration of an aminopeptidase activity in a filarial nematode glycoprotein. Molecular and Biochemical Parasitology 104: 11-23.

16. Lobos E, Weiss N, Karam M, Taylor HR, Ottesen EA, et al. (1991) An immunogenic *Onchocerca volvulus* antigen: A specific and early marker of infection. Science 251: 1603-1605.

17. Tree TIM, Gillespie AJ, Shepley KJ, Blaxter ML, Tuan RS, et al. (1995) Characterisation of an immunodominant glycoprotein antigen of *Onchocerca volvulus* with homologues in other filarial nematodes and *Caenorhabditis elegans*. Molecular and Biochemical Parasitology 69: 185-195.

18. Wu Y, Adam R, Williams SA, Bianco AE (1996) Chitinase genes expressed by infective larvae of the filarial nematodes, *Acanthocheilonema viteae* and *Onchocerca volvulus*. Molecular and Biochemical Parasitology 75: 207-219.

19. Hintz M, Schares G, Taubert A, Geyer R, Zahner H, et al. (1998) Juvenile female *Litomosoides sigmodontis* produce an excretory/secretory antigen (Juv-p120) highly modified with dimethylaminoethanol. Parasitology 117: 265-271.

20. Ghosh I, Eisinger SW, Raghavan N, Scott AL (1998) Thioredoxin peroxidases from *Brugia malayi*. Molecular and Biochemical Parasitology 91.
