# Supplementary material for: Stage- and Gender-Specific Proteomic Analysis of Brugia malayi Excretory-Secretory Products
Source: PLoS Negl Trop Dis. 2008 Oct 29;2(10):e326. doi: 10.1371/journal.pntd.0000326 (PMC2569413; doi:10.1371/journal.pntd.0000326)
Supplement: Alternative Language Abstract S1 — Translation of the Abstract into Spanish by Yovany Moreno (0.01 MB PDF) [file pntd.0000326.s005.pdf]

Análisis proteómico por estadío y por genero de los productos excretorios-secretorios de *Brugia malayi*

Resumen:

Introducción: Muchos de los mecanismos moleculares mediante los cuales los parásitos se establecen y logran protegerse de las respuestas inmunes de sus hospederos son aún desconocidos. Sin embargo, es aceptado a nivel general que muchos de estos procesos son mediados por productos liberados por el parasito, principalmente por proteínas. Una vez al interior de su hospedero, los nemátodos parasitarios se presentan en diferentes estadíos, a través de los cuales pueden establecerse en más de un compartimiento anatómico. En particular, es limitada la información acerca de la composición y variabilidad de los productos liberados durante los diferentes estadíos de su desarrollo y la contribución de estos en la supervivencia del parasito y la progresión de la infección.

Metodología/ hallazgos principales: Con el fin de obtener una valoración mas profunda en estos aspectos, se colectaron y analizaron a mediante 1D-SDS PAGE y LC/MS-MS los productos excretorios-secretorios (PES) de hembras adultas, machos adultos y microfilariae del nematodo filaria *Brugia malayi*, uno de los agentes etiológicos de la filariasis linfática humana. Este análisis proteómico permitió la identificación de 228 proteínas. Esta lista incluye 76 proteínas con función desconocida al igual que proteínas con el potencial de poseer propiedades immunoregulatorias tales como inhibidores de proteinasas, homólogos de citoquinas y proteínas de unión a carbohidratos. Consistente con esta observación, se asociaron diferentes perfiles de ontología de genes con los diferentes PES.

Conclusión/Impacto: Se presenta un análisis comparativo de las proteínas liberadas *in vitro* por las diferentes formas de un nemátodo parasitario que habitan un mismo hospedero. El catálogo de proteínas secretadas refleja diferentes procesos específicos de los estadíos y géneros analizados y diferentes estrategias de evasión del sistema inmune;

el análisis realizado provee claves significativas para comprender la contribución de cada forma del parásito en el establecimiento de su interacción con el hospedero.
