# Supplementary material for: Stage- and Gender-Specific Proteomic Analysis of Brugia malayi Excretory-Secretory Products
Source: PLoS Negl Trop Dis. 2008 Oct 29;2(10):e326. doi: 10.1371/journal.pntd.0000326 (PMC2569413; doi:10.1371/journal.pntd.0000326)
Supplement: Alternative Language Abstract S2 — Translation of the Abstract into French by Yovany Moreno and Timothy G. Geary (0.01 MB PDF) [file pntd.0000326.s006.pdf]

Analyse protéomique des produits excrétoires-sécrétoires de *Brugia malayi* en fonction du genre et du stade de développement

Abrégé

Introduction : Les mécanismes moléculaires par lesquels les parasites s'établissent et se protègent contre les réponses immunitaires de leurs hôtes sont méconnus. Néanmoins, il est accepté que plusieurs de ces processus sont modulés par des produits — généralement des protéines — libérés par le parasite. Une fois à l'intérieur de leur hôte, les nématodes parasitaires traversent différents stades de maturation au cours desquels ils peuvent s'établir dans plus d'un compartiment anatomique. Il existe toutefois peu d'informations au sujet de la composition et de la variabilité des produits libérés aux cours des diverses phases de développement. En outre, la contribution de ces produits à la survie et à la progression de l'infection demeure obscure.

Résultats principaux / méthodologie : L'objectif de cette étude était d'acquérir davantage de connaissances sur ces aspects de la biologie des nématodes parasitaires. Dans cette optique, nous avons recueilli et analysé par 1D-SDS PAGE et LC-MS/MS les produits excrétoires-sécrétoires (PES) de la femelle adulte, du mâle adulte et des microfilariae du nématode filarial *Brugia malayi*, un des agents étiologiques de la filariose lymphatique humaine. Cette analyse protéomique a permis l'identification de 228 protéines. Sur cette liste figurent 76 protéines annotées dont la fonction est inconnue, mais aussi des protéines ayant des propriétés immunorégulatrices potentielles, tels que des inhibiteurs de protéase, des homologues de cytokines et des protéines liant des hydrates de carbone. Les PES provenant des larves et des adultes diffèrent au niveau de leur composition. Seulement 32 des protéines identifiées sont communes aux 3 formes parasitaires analysées. Cette observation est corroborée par les profils d'ontologie de gène auxquels ont été associés les différents PES.

Conclusion/ Importance : Une analyse comparative des protéines libérées *in vitro* par les différentes formes d'un nématode parasitaire au sein d'un même hôte est présentée. Le catalogue de protéines sécrétées reflète les processus associés aux différents genres et stades de développement du parasite ainsi que les diverses stratégies d'évasion des réponses immunitaires de l'hôte. De plus, il fournit des indices importants au sujet de la contribution de chaque forme du parasite lors de l'établissement de l'interaction hôte-parasite.
